# High-yield production of protopanaxadiol from sugarcane molasses by metabolically engineered *Saccharomyces cerevisiae*

**DOI:** 10.1186/s12934-022-01949-4

**Published:** 2022-11-05

**Authors:** Yuan Zhu, Jianxiu Li, Longyun Peng, Lijun Meng, Mengxue Diao, Shuiyuan Jiang, Jianbin Li, Nengzhong Xie

**Affiliations:** 1 College of Light Industry and Food Engineering, Guangxi University, 100 Daxue Road, Nanning, 530004 China; 2 State Key Laboratory of Non-Food Biomass and Enzyme Technology, National Engineering Research Center for Non-Food Biorefinery, Guangxi Biomass Engineering Technology Research Center, Guangxi Academy of Sciences, 98 Daling Road, Nanning, 530007 China; 3 Guangxi Institute of Botany, Guangxi Zhuangzu Autonomous Region and the Chinese Academy of Sciences, Guilin, 541006 China

**Keywords:** Protopanaxadiol, Terpenoids, Synthetic biology, Sugarcane molasses, *Saccharomyces cerevisiae*

## Abstract

**Background:**

Ginsenosides are *Panax* plant-derived triterpenoid with wide applications in cardiovascular protection and immunity-boosting. However, the saponins content of *Panax* plants is fairly low, making it time-consuming and unsustainable by direct extraction. Protopanaxadiol (PPD) is a common precursor of dammarane-type saponins, and its sufficient supply is necessary for the efficient synthesis of ginsenoside.

**Results:**

In this study, a combinational strategy was used for the construction of an efficient yeast cell factory for PPD production. Firstly, a PPD-producing strain was successfully constructed by modular engineering in *Saccharomyces cerevisiae* BY4742 at the multi-copy sites. Then, the *INO2* gene, encoding a transcriptional activator of the phospholipid biosynthesis, was fine-tuned to promote the endoplasmic reticulum (ER) proliferation and improve the catalytic efficiency of ER-localized enzymes. To increase the metabolic flux of PPD, dynamic control, based on a carbon-source regulated promoter *P*_*HXT1*_, was introduced to repress the competition of sterols. Furthermore, the global transcription factor *UPC2-1* was introduced to sterol homeostasis and up-regulate the MVA pathway, and the resulting strain BY-V achieved a PPD production of 78.13 ± 0.38 mg/g DCW (563.60 ± 1.65 mg/L). Finally, sugarcane molasses was used as an inexpensive substrate for the first time in PPD synthesis. The PPD titers reached 1.55 ± 0.02 and 15.88 ± 0.65 g/L in shake flasks and a 5-L bioreactor, respectively. To the best of our knowledge, these results were new records on PPD production.

**Conclusion:**

The high-level of PPD production in this study and the successful comprehensive utilization of low-cost carbon source -sugarcane molassesindicate that the constructed yeast cell factory is an excellent candidate strain for the production of high-value-added PPD and its derivativeswith great industrial potential.

**Graphical Abstract:**

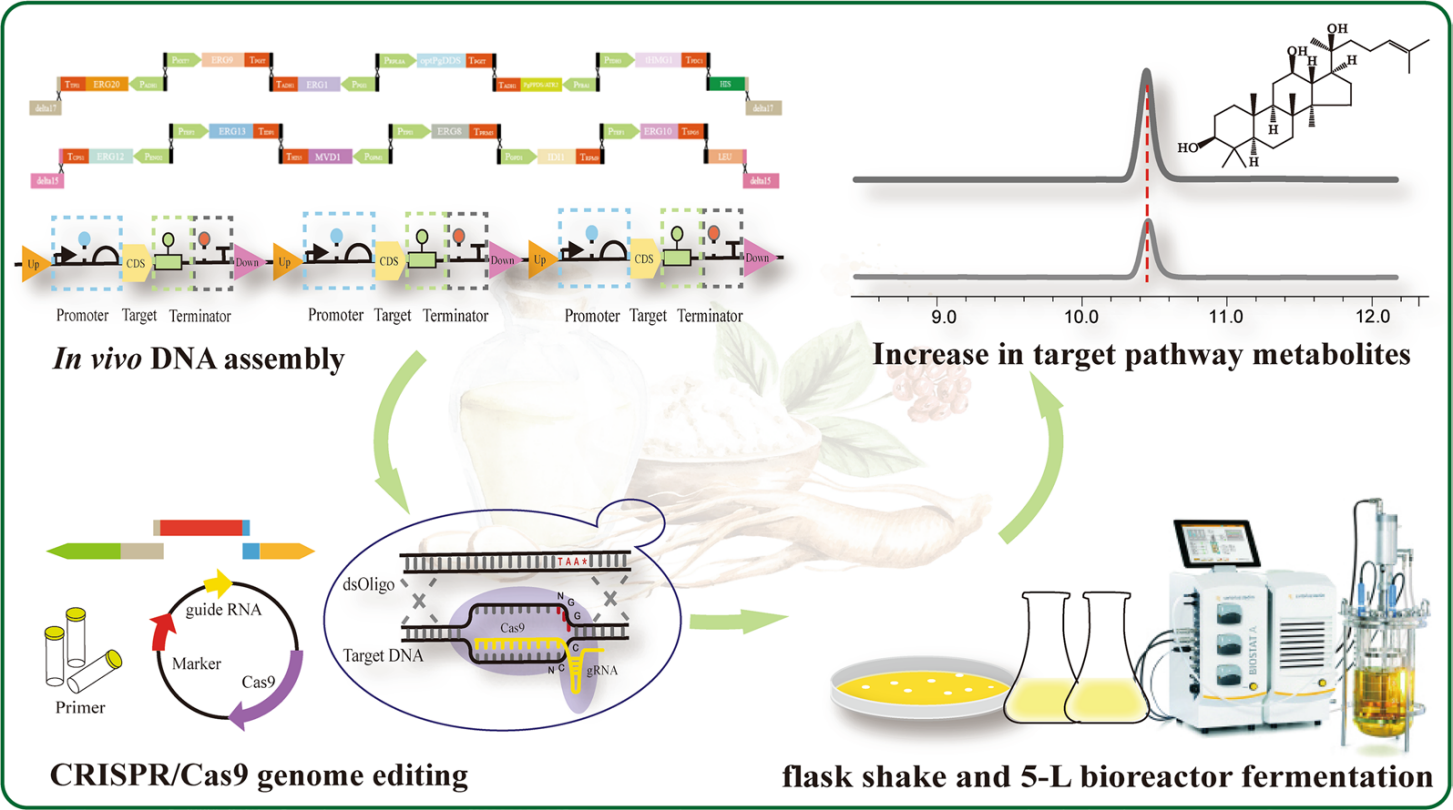

**Supplementary Information:**

The online version contains supplementary material available at 10.1186/s12934-022-01949-4.

## Background

*Panax ginseng* is a traditional Chinese medicine, widely used in Asia, Europe, and North America. Ginsenosides, the major bioactive components extracted from *Panax* plant, are a group of triterpenoids with diverse structural and pharmacological effects including alleviating fatigue and protecting the cardiovascular, endocrine, and immune systems [1–4]. However, the total ginsenosides contents in 5- to 7-year-old *P. ginseng* roots are approximately 2% g/g dry weight, and some rare ginsenosides accounts for less than 0.01%, making it time-consuming and unsustainable by direct extraction [5–7]. Moreover, due to the stereo-chemical complexity of ginsenosides, it is also challenging to synthesize by chemical methods [8].

Engineered microorganisms such as *Saccharomyces cerevisiae* and *Escherichia coli* provide an alternative approach for producing rare ginsenosides compounds to meet the continuously increasing market demand [9, 10]. Many active natural compounds, including lycopene [11], valencene [12], β-amyrin acetate [13], taxadiene and miltiradiene [14, 15], have been successfully produced through artificial microbial cell factories. Biosynthetic method is a green, sustainable and economical means to *de* *novo* synthesize natural compounds [16, 17].

Protopanaxadiol (PPD), the precursor of dammarane-type triterpene, is a promising antineoplastic and antidepressant drug candidate, which is hydroxylated from dammarenediol-II (DM-II) at the C12 position by *P. ginseng* PPD synthase (PgPPDS, also known as cytochrome P450 enzyme) [18, 19]. Many metabolic engineering strategies for PPD biosynthesis in *S. cerevisiae* have been developed, such as repression the competitive pathways, optimization of the cytochrome P450 oxidation system, endoplasmic reticulum (ER) amplification to facilitate PPD biosynthesis, etc. (Additional file 1: Table S1). Kim et al. expanded the ER in *S. cerevisiae* by overexpressing the key ER size regulatory factor *INO2*, which increased the production of squalene and PPD by 71-fold and 8-fold, respectively [20]. To overcome the poor coupling between PPDS and *Arabidopsis thaliana* cytochrome P450 reductase (ATR1), the PPDS-ATR1 fusion protein was introduced, and the PPD production increased significantly [21]. Then, Zhao et al. optimized the multi-genes pathway of PPD in *S. cerevisiae* by modular engineering strategies, of which the mevalonate (MVA) and acetyl-CoA pathway were up-regulated, and the sterol pathway was down-regulated. The PPD production of strain WLT-MVA5 reached 66.55 mg/g/OD_600_ in batch culture [22]. Wang et al. optimized the expression levels of MVA pathway genes and *PPDS* to increase the PPD metabolic flux in ZW04BY-RS. The PPD titer of ZW04BY-RS went up to 41.12 mg/g DCW in batch culture and 11.02 g/L in a 10-L bioreactor, which is the highest PPD production ever reported [23].

In this study, a PPD-producing strain was successfully constructed by modular engineering in *S. cerevisiae* BY4742 at the multi-copy sites. Then, the expression level of *INO2* was fine-tuned with strong promoters to promote the ER amplification and enhance the catalytic efficiency of cytochrome P450 enzymes. Furthermore, two competitive metabolic pathways were repressed by down-regulated lanosterol synthetase (*ERG7*) and phosphatidate phosphatase (*LPP1*). In addition, the global transcription factor *UPC2-1* was introduced to upregulate the MVA pathway. Finally, sugarcane molasses was used for the first time in PPD synthesis with restricted ethanol feeding. The PPD production of strain BY-V reached 1.55 ± 0.02 and 15.88 ± 0.65 g/L in fed-batch culture of shake flasks and a 5-L bioreactor respectively. This study paves the way for the development of an economical and efficient strategy for high-value-added natural compounds.

## Results and discussion

### Construction of PPD synthetic pathway in *S. cerevisiae*

**Fig. 1:**
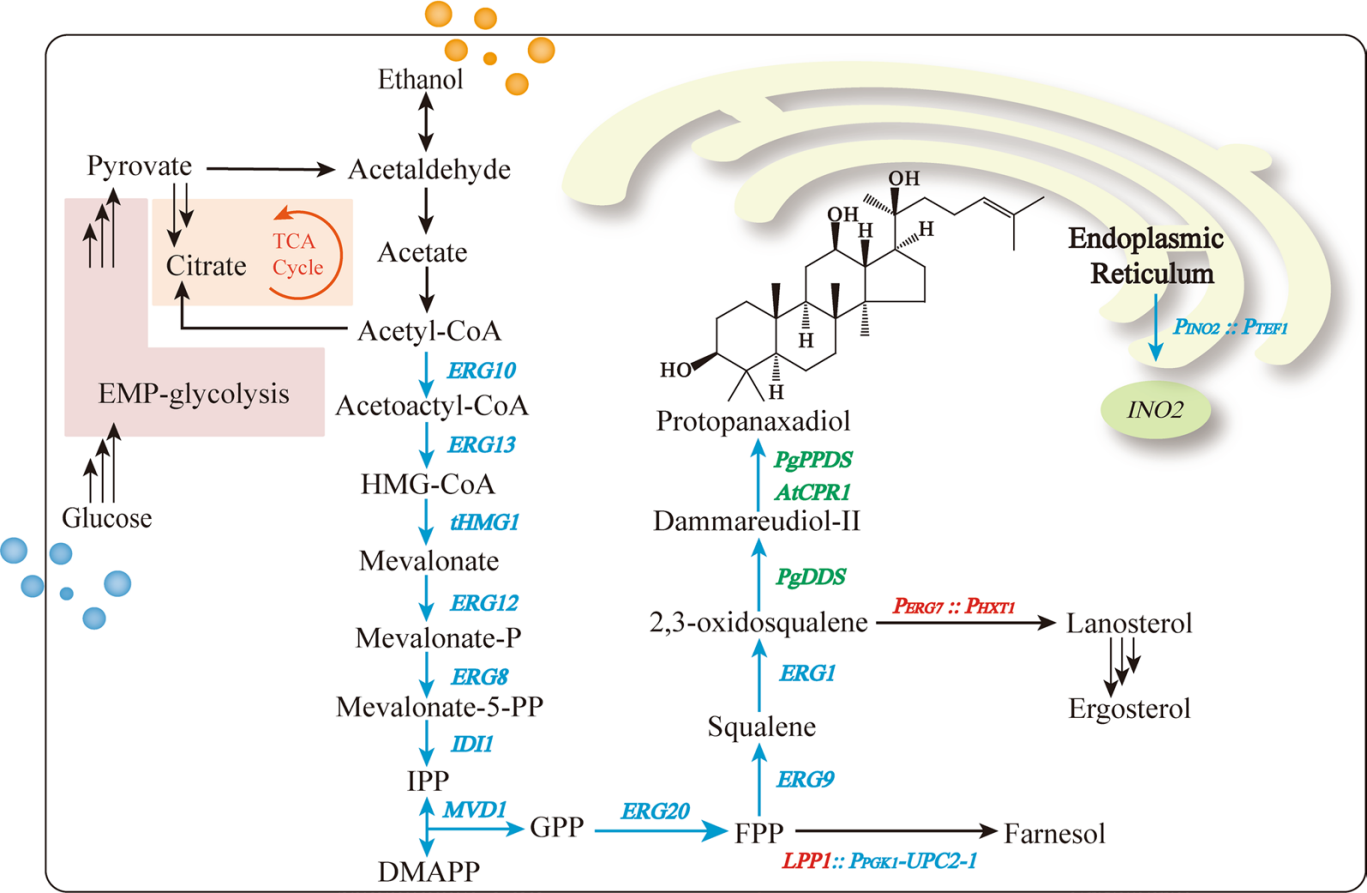
Biosynthesis pathways for PPD production in metabolically engineered *S. cerevisiae*. Single arrows represent one-step enzymatic conversions, and triple arrows represent multiple steps. Arrows marked in blue represent overexpressed steps. Genes indicated in blue and green font represent endogenous and heterologous, respectively. Genes indicated in red font were endogenous gene knocked out. *INO2*, a transcription factor for lipid biosynthesis; *ERG10*, acetyl-CoA C-acetyltransferase; *ERG13*, hydroxymethylglutaryl-CoA synthase; *tHMG1*, truncated HMG-CoA reductase; *ERG8*, phosphomevalonate kinase; *ERG12*, mevalonate kinase; *IDI1*, isopentenyl diphosphate δ-isomerase; *ERG20*, farnesyl diphosphate synthase; *ERG9*, squalene synthase; *ERG1*, 2,3-oxidosqualene synthase; *LPP1*, phosphatidate phosphatase; *PgDDS*, dammarenediol-II synthase from *Panax ginseng*; *PgPPDS* protopanaxadiol synthase from *Panax ginseng*; *AtCPR1,* NADPH-cytochrome P450 reductase from *Arabidopsis thaliana*

**Fig. 2:**
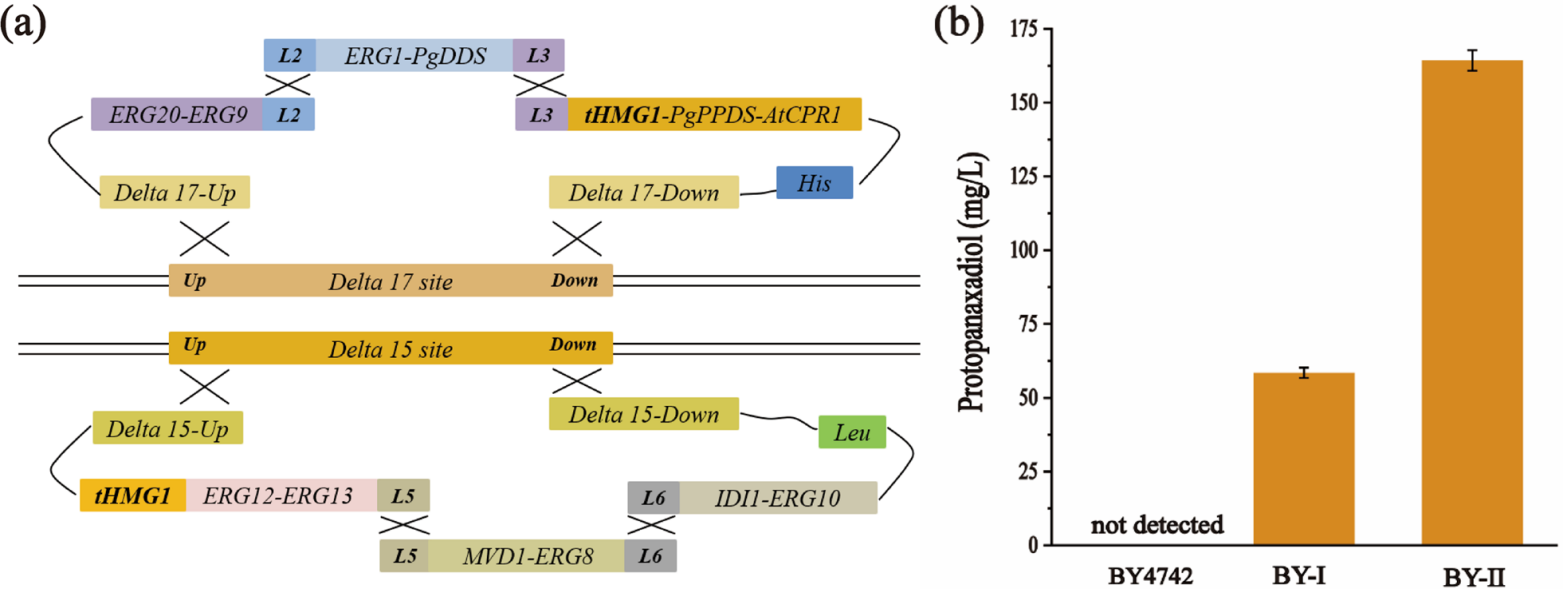
Construction of PPD-producing strain. **a** Engineering strain construction using* in vivo* DNA assembly. **b** PPD production of engineering strains. The error bars indicate three biological replicates

PPD is a common precursor of PPD-type saponins, and its accumulation is essential to the production of ginseng metabolites [24]. In *S. cerevisiae*, the glycolytic flux is directed towards ethanol due to the Crabtree effect during cell growth on glucose [25]. Then, ethanol was converted to acetaldehyde through cytosolic acetaldehyde dehydrogenase. Acetyl-CoA is further oxidized from acetate, which is derived from acetaldehyde [26]. The PPD synthesis from acetyl-CoA requires 13 enzymatic steps (Fig. 1). PPD biosynthesis-related enzymes were thus divided into two expression cassettes, as shown in Fig. 2a, and *delta17* and *delta15* were chosen for multi-copy integration [27, 28]. The first cassette includes seven genes, namely, *ERG9*, *ERG20*, *ERG1*, *PgDDS*, *PgPPDS*, *AtCPR1*, and *tHMG1*, which were integrated into the *delta17* multi-copy site of BY4742 to construct the PPD synthetic pathway. Transformants were screened using CM-His medium and further verified through PCR amplification.

The heterologous genes *PgDDS*, *PgPPDS* and *AtCPR1*, which encode dammarenediol-II synthase (DDS), protopanaxadiol synthase (PPDS) and NADPH-cytochrome P450 reductase (CPR1), respectively, are essential for PPD synthesize from 2,3-oxidosqualene. Because the multiple integrations occurred randomly with a low probability (about 1–10%), there was a huge range of PPD production among the clones screened [29]. A total of 192 positive colonies were examined via HPLC analysis after shake flask fermentation for 72 h (Additional file 1: Fig. S1a). The PPD-producing strain named B-C9 had a PPD yield of 2.21 ± 0.45 mg/g DCW (10.93 ± 0.89 mg/L), while strain BY-I achieved a PPD yield of 11.32 ± 0.45 mg/g DCW (58.43 ± 1.76 mg/L). The copy numbers and transcription levels of two heterologous genes (*PgPPDS* and *AtCPR1*) and two endogenous genes (*tHMG1* and *ERG9*) of the first cassette were detected using real-time fluorescence quantitative PCR (RT-qPCR). In comparison with B-C9, the copy number and RNA transcription levels of *ERG9*, *PgPPDS*, *AtCPR1*, and *tHMG1* in BY-I have risen to varying degrees, which makes it an outlier (Additional file 1: Fig. S2).

Next, the second cassette composed of *ERG8*, *ERG12*, *ERG13*, *MVD1*, *IDI1, ERG10*, and *tHMG1* was integrated into the *delta15* multi-copy site of BY-I to strengthen the transformation of acetyl-CoA into isopentenyl pyrophosphate (IPP) and dimethylallyl pyrophosphate (DMAPP), which were the isoprenoid building blocks. Considering that HMG1 is the rate-limiting enzyme of the mevalonate pathway (MVA pathway), *tHMG1* was integrated again to increase HMG-CoA flux [30]. One hundred and fifty-three positive colonies were verified using HPLC (Additional file 1: Fig. S1b). The PPD yield of strain BY-II exhibited a prominent improvement and achieved 33.23 ± 0.26 mg/g DCW (164.30 ± 3.48 mg/L), which was 2.94 times higher than that of BY-I (Fig. 2b). The copy numbers and transcription levels of four endogenous genes (*tHMG1, ERG8, ERG10 and IDI1*) of the second cassette were detected. And the relative transcription levels of *tHMG1*, *ERG8*, *ERG10* and *IDI1* in BY-II were 1.61, 2.07, 2.17, and 2.01 times higher than that of BY-I (P < 0.01) (Additional file 1: Fig. S2), which were consist with PPD production.

### Enhancing PPD production by engineering endoplasmic reticulum (ER)

**Fig. 3:**
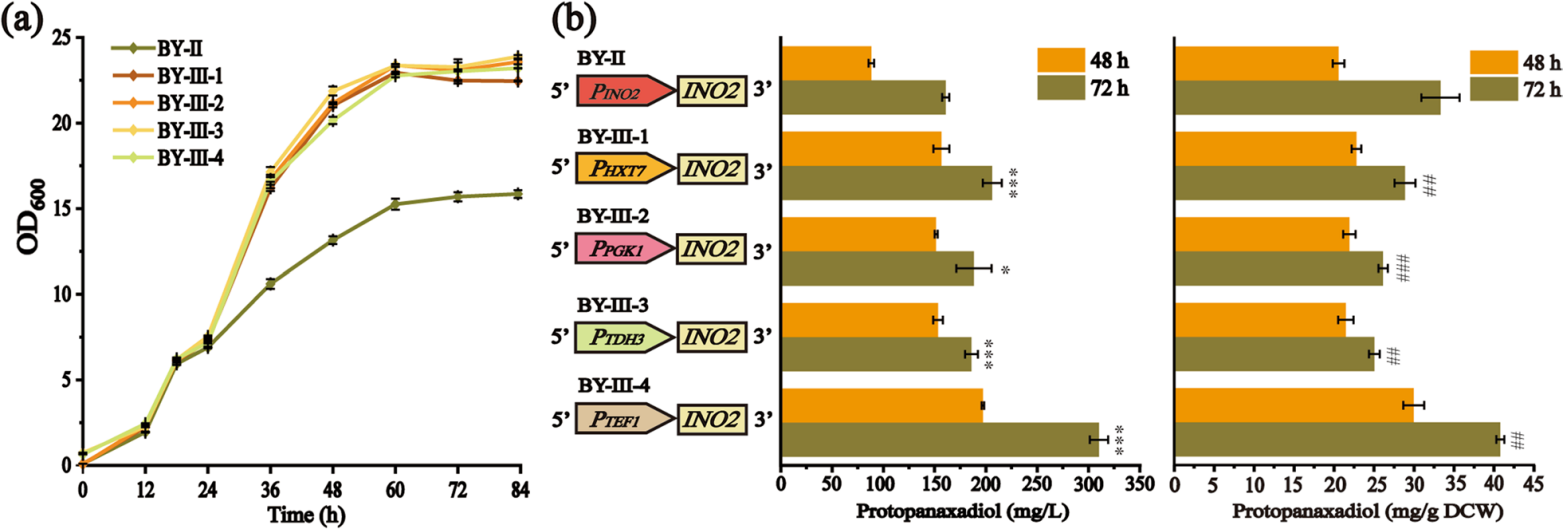
Comparison of (**a**) cell growth and (**b**) PPD production of engineered strains in 250-mL shake flasks with 50 mL YPD medium. The endogenous promoter of *INO2* gene of BY-II is replaced by *P*_*HXT7*_, *P*_*PGK1*_, *P*_*TDH3*_, and *P*_*TEF1*_ to construct strains BY-III-1, BY-III-2, BY-III-3, and BY-III-4, respectively. Asterisks or pounds denote statistically significant differences from BY-II as determined by a Student’s t-test (*P < 0.05; **P < 0.01; ***P < 0.001; ^#^*P* < 0.05; ^##^*P* < 0.01; ^*###*^*P* < 0.001). All data are presented as mean ± standard deviation of biological triplicates

*S. cerevisiae* is an ideal platform for heterologous biosynthesis of triterpenoids [31]. However, the low catalytic efficiency of cytochrome P450 enzymes (P450s), which require NADPH-cytochrome P450 reductases (CPR) to provide electrons, was the primary challenge for terpenoids synthesis [32]. ER proliferation could enhance the insertion and retention of the P450 reductase in the ER membrane to reach a high-level catalytic efficiency of membrane-localized P450s [33]. The key ER regulatory factor *INO2*, together with *INO4* and *OPI1*, are the primary ER responsive elements of *S. cerevisiae*, which constitute an auto-regulatory phospholipid biosynthesis system[34]. It has been reported that overexpression of *INO2* for ER expansion could drive ER sheets proliferation, alleviate stress and improve the cell viability [35]. In the present study, *INO2* was overexpressed through promoter swapping. Four strong promoters, namely, *P*_*HXT7*_, *P*_*PGK1*_, *P*_*TDH3*_, and *P*_*TEF1*_, were selected to replace the *INO2* endogenous promoter of BY-II, resulting in BY-III-1, BY-III-2, BY-III-3, and BY-III-4, respectively. As seen in Fig. 3a, the cell growth of BY-III strains far surpassed that of BY-II after 24 h. The PPD production of BY-III-1, BY-III-2, BY-III-3, and BY-III-4 at 48 h reached 156.75 ± 7.89, 151.55 ± 1.44, 153.43 ± 4.77, and 197.26 ± 1.14 mg/L, respectively, which equaled or even exceeded that of BY-II at 72 h. Notably, strain BY-III-4 exhibited a surprised PPD yield of 40.79 ± 0.30 mg/g DCW (310.35 ± 8.96 mg/L) at 72 h, which increased 1.89 times than that of BY-II (164.30 ± 3.48 mg/L) (P < 0.001) (Fig. 3b and Table 1). Kim et al. previously reported that overexpressing *INO2* could expand the ER, thus improve the capacity to synthesize ER-associated proteins and cytochrome P450-mediated PPD, and increase available space to accommodate them [20]. In our study, the cell growth and PPD production of BY-III strains are significantly improved by up-regulation *INO2* possibly due to efficient localization of cytochrome P450 in an expanded ER as a possible mechanism, which is consistent with Kim’s studies [36].

**Table 1:** PPD production of engineered strains in batch culture for 72 h

Strain	PPD (mg/g DCW)	Fold of BY-II	PPD (mg/L)	Fold of BY-II	PPD/sugar (mg/g)	DM-II (mg/L)
BY4742	NA^a^	–	NA	–	–	NA
BY-I	11.32 ± 0.45^b^	–	58.43 ± 1.76	–	1.95 ± 0.51	7.43 ± 2.11
BY-II	33.23 ± 0.26	1.00	164.30 ± 3.48	1.00	5.48 ± 0.43	15.11 ± 0.32
BY-III-1	28.02 ± 0.02	0.84	206.54 ± 13.81	1.34	6.88 ± 0.58	28.68 ± 2.48
BY-III-2	26.69 ± 0.27	0.81	203.07 ± 2.78	1.24	6.77 ± 0.23	30.66 ± 1.24
BY-III-3	25.17 ± 0.05	0.76	194.23 ± 8.74	1.18	6.47 ± 0.59	28.28 ± 1.17
BY-III-4	40.79 ± 0.30	1.22	310.35 ± 8.96	1.89	10.35 ± 0.31	39.68 ± 4.29
BY-III-5	57.25 ± 0.25	1.72	412.13 ± 1.93	2.51	13.74 ± 0.46	25.91 ± 0.32
BY-IV	59.46 ± 0.57	1.79	414.75 ± 6.11	2.52	13.83 ± 0.12	177.64 ± 14.00
BY-V	78.13 ± 0.38	2.35	563.60 ± 1.65	3.43	18.79 ± 0.23	66.91 ± 7.38

^a^NA: Not applicable

^b^All data are presented as mean ± standard deviation of biological triplicates

### Improving PPD production by metabolic pathway optimization

**Fig. 4:**
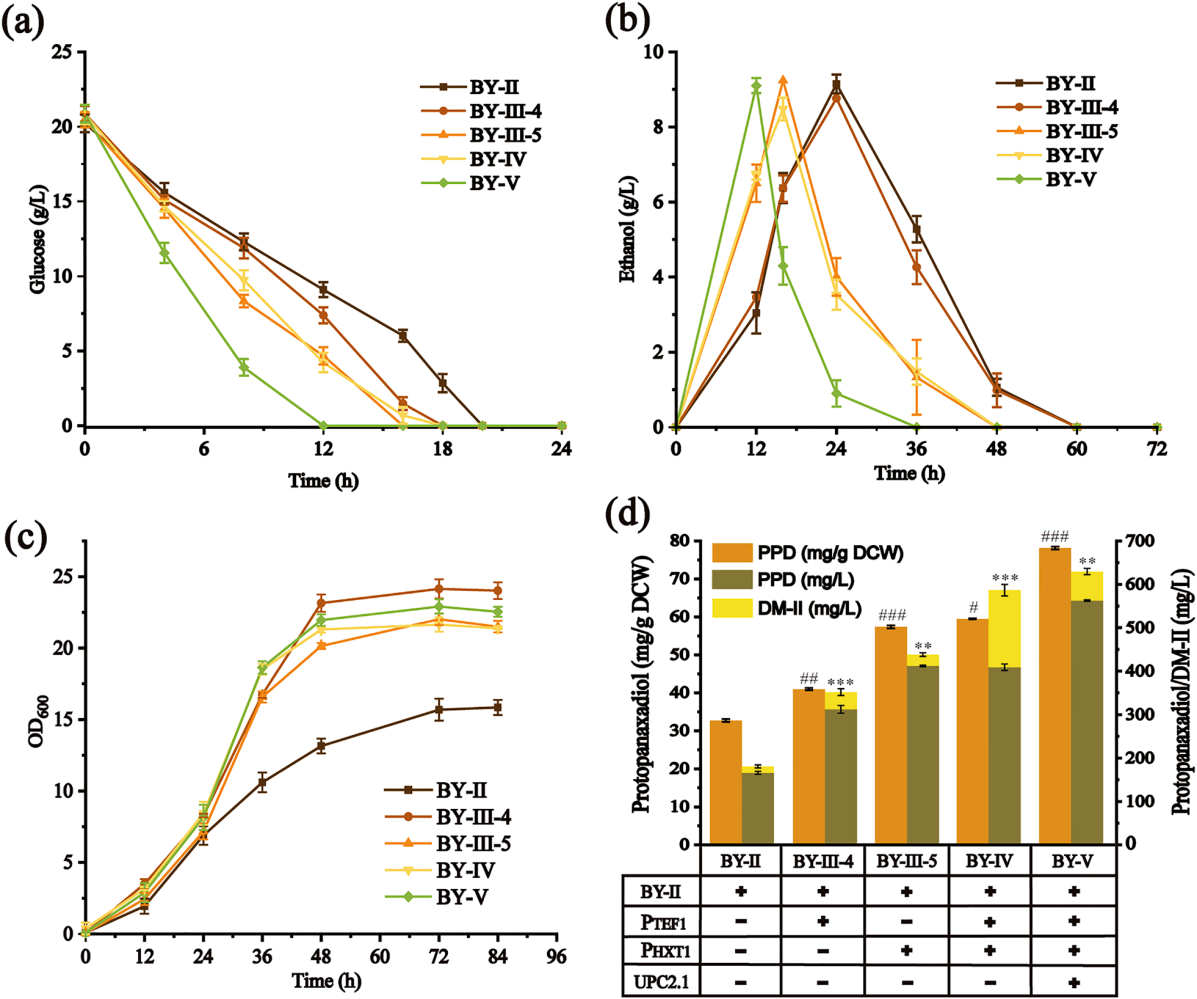
Effect of metabolic pathway optimization on PPD production. (**a**) Residual glucose, (**b**) ethanol consumption, (**c**) cell growth, (**d**) DM-II and PPD titers of PPD-producing strains. Data are presented as the means of three replicates, and bars represent the standard deviations. Asterisks or pounds denote statistically significant differences from BY-II as determined by a Student’s t-test (*P < 0.05; **P < 0.01; ***P < 0.001; ^#^*P* < 0.05; ^##^*P* < 0.01; ^*###*^*P* < 0.001)

For PPD biosynthesis, the lanosterol pathway is a competing pathway [37]. It has been reported that down-regulation of lanosterol synthetase (*ERG7*) expression can increase the metabolic flux of target terpenoid [38, 39]. However, as one of the inherent components of cell membrane, lanosterol is essential for the normal growth of *S. cerevisiae*, thus cannot be knocked out [40, 41]. In the current work, dynamic control, based on a carbon-source regulated promoter *P*_*HXT1*_, was introduced to relieve the competition between cell-growth and PPD production associated processes. Maury et al. previously characterized the *P*_*HXT1*_ promoter in *S. cerevisiae* via transcriptional analysis, which was high expressed in glucose-excess and low expressed in glucose-limiting conditions [42]. Owning to the glucose-sensing toggle switch of *P*_*HXT1*_, the cell-growth of BY-III-5 was divided in a glucose growth phase and an ethanol growth phase, and BY-III-5 was conferred a significant increase of PPD production, with a PPD yield of 57.25±0.25 mg/g DCW (412.13 ± 1.93 mg/L) at 72 h, which is 2.51 times higher than that of BY-II (Fig. 4 and Table 1). To further test the efficiency of *P*_*HXT1*_, the endogenous promoter of *ERG7* in BY-III-4 was replaced by *P*_*HXT1*_, resulting in BY-IV. Although a lower growth in logarithmic phase was observed in BY-IV compared to BY-III-4, the metabolic flux of DM-II in BY-IV was promoted conspicuously, showing a DM-II accumulation of 177.64 ± 13.39 mg/L. It is possible that *ERG7* was induced in the presence of glucose at the early stage of cell growth and repressed after the depletion of glucose, which boosted the synthesis of DM-II and impaired the synthesis of lanosterol [43].

Maintaining the sterol homeostasis is of paramount importance for fungi growth and metabolism. The sterol-regulating transcription factor *UPC2* plays an essential role in the sterol homeostasis of *S. cerevisiae* by upregulating MVA pathway, and has been successfully used to enhance terpenoids production [44, 45]. To further improve PPD production, *UPC2.1*, the G888D mutant of *UPC2*, was introduced. Meanwhile, the phosphatidate phosphatase (*LPP1*) was knocked out to diminish the metabolic flux of farnesol [46]. Hence, the *P*_*PGK1*_-*UPC2.1*-*T*_*ADH1*_ expression cassette was knocked into the *LPP1* locus of BY-IV by CRISPR/*Cas9*, resulting in BY-V. As illustrated in Fig. 4, the yield of PPD by strain BY-V was further increased. Moreover, the DM-II of BY-V declined to 66.91 ± 7.38 mg/L, and the PPD yield went up to 78.13 ± 0.38 mg/g DCW (563.06 ± 1.65 mg/L) (P < 0.01), which was a new record to the best of our knowledge.

### PPD production in shake flasks

**Fig. 5:**
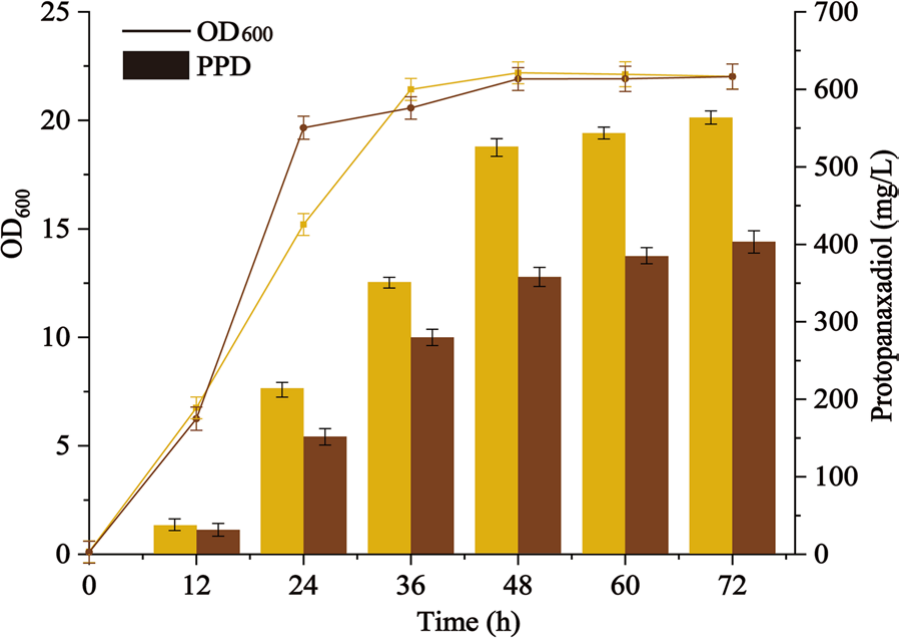
Cell growth and PPD production of BY-V in different carbon sources. Curves and bars marked in yellow and brown represent BY-V cultivated with glucose and molasses, respectively. Data are presented as the means of three replicates, and bars represent the standard deviations

As a major by-product of sugar manufacturing process, molasses contains approximately 50% fermentable sugars and a small number of nitrogenous compounds, inorganic salts, and trace elements, which are the essential nutrients for growth and biosynthesis of *S. cerevisiae* [47]*.* The shake flask fermentation was conducted with an initial molasses concentration of 40 g/L. As shown in Fig. 5, the cell growth of strain BY-V in molasses was faster at the logarithmic phase, and closed at stationary phase, compared with that of YPD. However, the PPD titer and PPD yield (PPD/sugar) in molasses were 402.22 ± 7.39 mg/L and 13.11 ± 0.16 mg/g, respectively, at 72 h, which were just 71.37% of those in YPD medium.

**Fig. 6:**
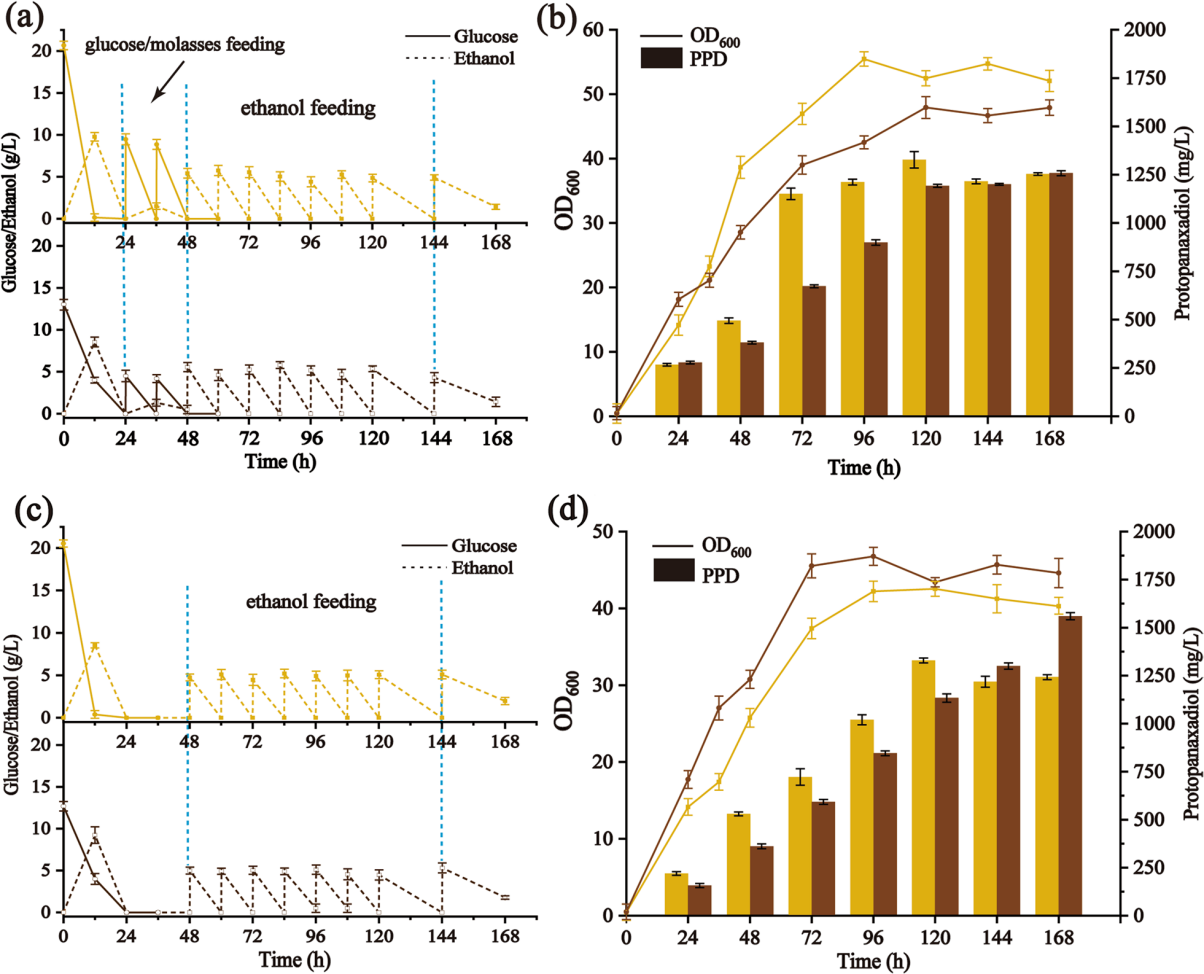
Effect of different feeding strategies on cell growth and PPD production. (**a**) Glucose/ethanol consumption, and (**b**) cell growth and PPD production of BY-V by two-stage feeding. (**c**) Glucose/ethanol consumption, and (**d**) cell growth and PPD production of BY-V by restricted ethanol feeding. The yellow curves and bars represent fermentation in glucose, and brown curves and bars represent fermentation in molasses. Data are presented as the means of three replicates, and bars represent the standard deviations

To further improve PPD titer, fed-batch fermentation was carried out. The potential of BY-V for PPD production was firstly investigated by restricted glucose/molasses feeding strategy (Table 2 and Additional file 1: Fig. S3). The production of PPD with glucose and molasses feeding achieved 841.09 ± 2.16 mg/L and 556.26 ± 4.90 mg/L, respectively. The lower PPD titer in molasses might be caused by the large quantities of ash and metal ions in molasses, which inhibit the synthesis of target compounds [47]. Then, two-stage feeding strategy was conducted. The initial concentrations of glucose and molasses in YPD medium were 20 and 40 g/L (containing approximately 20 g/L of fermentable sugars), respectively. In the early stage, glucose and molasses were fed to improve cell growth, respectively. After 48 h, ethanol (99.7%, v/v) was added at intervals to facilitate PPD accumulation. As shown in Fig. 6a, the two-stage feeding strategy resulted in high cell biomass. The OD_600_ of BY-V achieved 48.62 in molasses-ethanol, which is 2.12 and 1.81 times higher than that of batch and fed-batch in molasses, respectively. Moreover, the PPD titer went up to 1.25 ± 0.01 g/L after 168 h, with a PPD yield [PPD/(sugar + ethanol)] of 15.63 ± 0.83 mg/g (Fig. 6b and Table 2).

**Table 2:** PPD production of strain BY-V in fed-batch culture

Carbon source	Feeding strategy	PPD^a^ (mg/g DCW)	PPD (mg/L)	[PPD/(sugar + ethanol)] (mg/g)	Time (h)
Glucose	NA^b^	78.13 ± 0.38^c^	563.60 ± 1.65	18.79 ± 0.23	72
Molasses	NA	53.04 ± 0.30	402.22 ± 7.39	13.11 ± 0.16	72
Glucose	G + N^d^	41.29 ± 0.78	841.09 ± 2.16	8.14 ± 0.56	96
Molasses	M + N	64.33 ± 0.54	556.26 ± 4.90	11.13 ± 0.44	120
Glucose	G + E + N	77.01 ± 0.17	1320.69 ± 42.51	14.67 ± 0.68	120
Molasses	M + E + N	79.92 ± 0.19	1251.56 ± 12.54	15.63 ± 0.83	168
Glucose	E + N	95.15 ± 0.96	1323.22 ± 13.13	19.41 ± 0.75	120
Molasses	E + N	106.55 ± 0.91	1553.68 ± 18.72	22.79 ± 1.06	168

^a^PPD yields represented the total amounts of intracellular and extracellular extraction

^b^NA: Not applicable

^c^All data are presented as mean ± standard deviation of biological triplicates

^d^G, N, M and E represented glucose, nitrogen, molasses and ethanol, respectively

Ethanol was commonly used in yeast fermentation for terpenoids accumulation [48]. Zhang et al. has reported that 138.80 mg/L of β-amyrin production was achieved using pure ethanol feeding [44]. In this study, ethanol was fed as the sole carbon source after glucose/molasses depletion (Fig. 6c). The OD_600_ of BY-V was 46.94, slightly lower than that in two-stage feeding with molasses-ethanol. Surprisingly, 1.55 ± 0.02 g/L (106.55 ± 0.91 mg/g DCW) of PPD was accumulated at 168 h, which is 2.79 and 1.24 times higher than that of molasses feeding and two-stage feeding with molasses-ethanol. Moreover, the PPD yield of restricted ethanol feeding strategy went up to 19.41 ± 0.75 mg/g and 22.79 ± 1.06 mg/g, respectively (Fig. 6d and Table 2). These results demonstrated that although ethanol served as a non-fermentable carbon source and might hinder the growth of engineering chassis, it facilitated the synthesis of PPD via acetyl-CoA pathway directly rather than by the complex glycolytic pathway [49].

### PPD production in a 5-L bioreactor

**Fig. 7:**
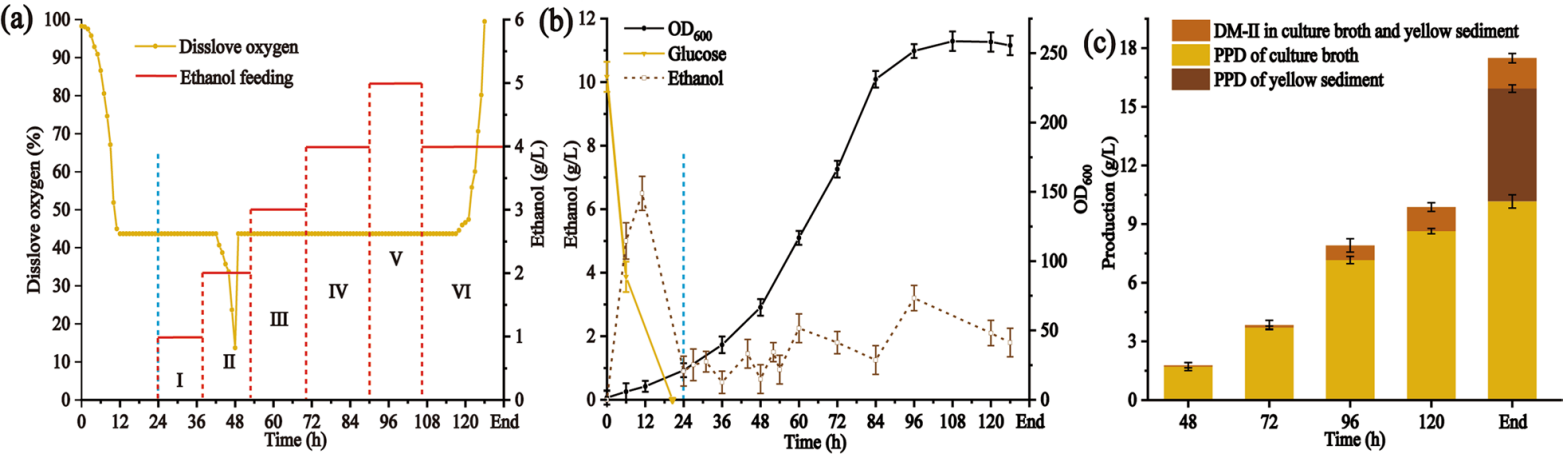
Fed-batch fermentation of BY-V in a 5-L bioreactor. (**a**) Dissolve oxygen, ethanol feeding, and (**b**) cell density, glucose/ethanol consumption of strain BY-V. (**c**) DM-II and PPD production from the fermented broth and faint yellow sediment. Stage I, II, III, IV, V, VI represent 1, 2, 3, 4, 5, 4 g/L ethanol fed at intervals to control the concentration of ethanol at the range of 1–5 g/L. A total of 230 mL ammonia was added to maintain the pH at 5.5. Error bars indicate standard deviations (*n* = 3)

To evaluate the performance of the PPD production of strain BY-V in high-density culture, a 5-L bioreactor with 1.5 L of synthetic medium was employed. Ethanol was fed at intervals to control the ethanol concentration in the range of 1–5 g/L. The ethanol metabolism results the accumulation of NADH, which is regenerated by oxidative phosphorylation and consequently consumes large amounts of oxygen [50]. Hence, adequate oxygen supplement is needed to promote cell growth and PPD synthesis. To maintain the dissolve oxygen (DO) of fermentation broth at 40%, pure oxygen was supplied when the cell growth of BY-V entered logarithmic phase (about 48 h) (Fig. 7a and b). Then, the strain entered stationary phase at 96 h, and attained a maximum OD_600_ of 262.14 at 108 h. The PPD of culture broth was continued to accumulate with a PPD titer of 8.63 ± 0.13 g/L (100.82 ± 0.42 mg/g DCW) at 120 h (Fig. 7c and Table 3). Zhao et al. reported that a large amount of PPD was secreted to extracellular space and adhered to the stainless pipe and the inner tank wall [51]. Notably, PPD mainly showed intracellular accumulation (10.18 ± 0.35 g/L) with sugarcane molasses as the initial carbon source in a 5-L bioreactor. As compared with glucose, sugarcane molasses generates less foam and adhered sediment, which increases oxygen transferring and facilitates downstream pretreatment and separation [23, 51]. In our study, the total PPD production, including fermented broth and faint yellow sediment, attained 15.88 ± 0.65 g/L (188.50 ± 0.56 mg/g DCW) at the end of the fermentation (Fig. 7c, Table 3 and Additional file 1: Fig. S5). The PPD titer was a new record and 1.44 times higher than that of ZW04BY-RS, reported by Wang et al. [23]. This result indicates that using molasses as the cheap carbon source with ethanol feeding is an effective strategy for PPD production.

**Table 3:** PPD and DM-II production of strain BY-V in 5-L bioreactor

Time (h)	PPD^a^ (mg/g DCW)	PPD (g/L)	[PPD/(sugar + ethanol)] (mg/g)	DM-II (g/L)	DM-II/PPD (%)
48	78.55 ± 0.36^b^	1.71 ± 0.20	12.10 ± 0.32	0.07 ± 0.01	2.08 ± 0.21
72	81.86 ± 0.77	3.71 ± 0.07	14.38 ± 0.45	0.13 ± 0.04	3.35 ± 0.35
96	87.06 ± 0.45	7.16 ± 0.18	15.91 ± 0.74	0.75 ± 0.04	9.46 ± 0.43
120	100.82 ± 0.42	8.64 ± 0.13	15.16 ± 0.56	1.23 ± 0.02	12.47 ± 0.25
126	188.50 ± 0.56	15.88 ± 0.65	25.05 ± 0.47	2.19 ± 0.04	13.02 ± 0.36

^a^PPD yields represented the total amounts of intracellular and extracellular extraction

^b^All data are presented as mean ± standard deviation of biological triplicates

## Conclusion

In this study, we adopted various strategies, including metabolic engineering, promoter engineering, ER engineering, and lanosterol pathway down-regulation to improve heterogenous PPD biosynthesis of *S. cerevisiae*. The strain BY-V, engineered by combinational strategies, exhibited a prominent improvement in PPD biosynthesis and achieved a PPD yield of 78.13 ± 0.38 mg/g DCW (563.60 ± 1.65 mg/L), which was a new record to the best of our knowledge. Thereafter, sugar molasses, a major by-product of sugar manufacturing process, was first used for PPD synthesis. After the optimization of fermentation process, the PPD titer (1.55 ± 0.02 g/L and 106.55 ± 0.91 mg/g DCW) from sugar molasses with restrict ethanol feeding in shake flasks was much higher than that of glucose (1.32 ± 0.01 g/L and 95.15 ± 0.96 mg/g DCW). In a 5-L bioreactor, the total PPD production (in both the culture broth and the yellow sediment) attained 15.88 ± 0.65 g/L and 188.50 ± 0.56 mg/g DCW at the end of the fermentation. This study provides a reference for the comprehensive utilization of molasses via a low cost and environment-friendly approach, and also an example for the biosynthesis of high-value-added natural compounds.

## Methods

### Strains and medium

*S. cerevisiae* BY4742 (*MATα*, *his3*Δ*1*, *leu2*Δ*0*, *lys2*Δ*0*, *ura3*Δ*0*) obtained from American Type Culture Collection (Manassas, VA, USA) was used as the parent strain. Yeast was grown in YPD medium (20 g/L glucose, 20 g/L peptone, and 10 g/L yeast extract) or CM medium (20 g/L glucose, 6.7 g/L yeast nitrogen base without amino acids, and 0.8 g/L dropout powder minus appropriate amino acids) at 30 °C. *E. coli* Trans 5α (TransGen Biotech, Beijing, China), cultivated at 37 °C in LB medium, was used for plasmid amplification. All of the strains and plasmids used in this study are listed in Table 4 and Additional file 1: Table S2. Molasses used in this study was purchased from Guangxi Sugar Industry Group Co., Ltd (Nanning, China).

**Table 4:** Strains used in this study

Strain	Genotype	Reference
*E. coli* DH5α	F- φ80*lac* ZΔM15, Δ(*lac*ZYA-*argF*) U169, *deoR*, *recA1*, *endA1*, *hsdR17*(rk^−^mk^+^) supE44, *λ*-, *thi*-1, *gyrA96*, *relA1 phoA*	Novagen
BY4742	*MATα his3*Δ*1 leu2*Δ*0 lys2*Δ*0 ura3*Δ*0*	ATCC
BY-I	*P*_*ADH1*_-*ERG20-T*_*TPI1*_, *P*_*HXT7*_*-ERG9-T*_*PGIT*_, *P*_*PGI*_-*ERG1*-*T*_*ADH*_, *P*_*RPL8A*_*-PgDS-T*_*CYC1*_, *P*_*ADH1*_-*CYP716A47-46ATR1-T*_*FBA1*_, *P*_*TDH3*_*-tHMG1-T*_*PDC1*_ cassettes and *HIS5* marker gene were integrated into *delta17* site of BY4742	This study
BY-II	*P*_*ENO2*_*-ERG12-T*_*CPS1*_, *P*_*TEF2*_*-ERG13-T*_*IDP1*_, *P*_*GPM1*_-*MVD1-T*_*HIS5*_, *P*_*TPI1*-_*ERG8-T*_*PRM5*_, *P*_*GDP1*_*-IDI1-T*_*PRM9*_, *P*_*TDH3*_-*tHMG1-T*_*TDH2*_, *P*_*TEF1*_*-ERG10-T*_*SPG5*_ cassettes and *LEU2* marker gene were integrated into *delta15* site of BY-I	This study
BY-III-1	BY-II*, P*_*INO2*_::*P*_*HXT7*_	This study
BY-III-2	BY-II*, P*_*INO2*_::*P*_*PGK1*_	This study
BY-III-3	BY-II*, P*_*INO2*_::*P*_*TDH3*_	This study
BY-III-4	BY-II*, P*_*INO2*_::*P*_*TEF1*_	This study
BY-III-5	BY-II*, P*_*ERG7*_::*P*_*HXT1*_	This study
BY-IV	BY-III-4*, P*_*ERG7*_::*P*_*HXT1*_	This study
BY-V	BY-IV, *LPP1*::*P*_*PGK1*_*-UPC2.1*	This study

### Plasmids construction

The sequences of *PgDDS*, *PgPPDS*, and *AtCPR1* (GenBank Accession Nos. ACZ71036.1, AEY75213.1, and AIC73829.1) were codon optimized and synthesized by Wuhan Gene Create Biological Engineering Co., Ltd. (Wuhan, China). Then, these synthesized DNA fragments were cloned into pUC57, resulting in pUC57-*PgDDS* and pUC57-*PgPPDS*/*AtCPR1*. Promoters, terminators, genes, and homologous arms were amplified from the genome of BY4742 via PCR with specific primers (Additional file 1: Table S3). The selection marker (i.e., *HIS*, *LEU*, and *URA3*) were amplified from plasmid PYES3-CT. N-terminally truncated *HMG-CoA* reductase (*tHMG1*) was artificially synthesized. All fragments were purified using Gel Recovery Kit (GenStar, Beijing, China). Promoters, terminators, and genes were spliced by overlap extension PCR to synthesize expression cassettes. The plasmid DNA including the target fragments (*P*_*TEF1*_, *P*_*HXT1*_, and *P*_*PGK1*_-*UPC2.1*-*T*_*ADH1*_) were sequenced by Sangon Biotech Co., Ltd. (Sangon Biotech, Shanghai, China). Finally, the fragments were co-transformed into yeast using LiAc/ssDNA method.

### Quantification of genes copy numbers and RNA transcription level

Genomic DNA was extracted using TIANamp Yeast DNA Kit (Tiangen Biotech, Beijing, China), and RNA was extracted using Trizol (Invitrogen, Carlsbad, USA) following product manuals. cDNA was obtained by reverse transcription-polymerase chain reaction using a Prime Script One Step RT-PCR kit (Takara, Beijing, China). Quantitative real-time PCR (RT-qPCR) was performed using Prime Script RT reagent kit with gDNA eraser (Takara, Beijing, China) [52]. Primers used for RT-qPCR were listed in Additional file 1: Table S4.

### CRISPR/*Cas9* gene editing in *S. cerevisiae*

gRNA sequences with 100% specificity to other genomes was obtained using online gene editing tools, and target sequences with the highest scores were selected [53]. All gRNA target sequences used in this study were listed in Additional file 1: Table S5. The plasmid skeleton of pCAS-*RNR2p*-*Cas9*-*CYC1t* was amplified with pCas9-F/R primers (Additional file 1: Fig. S4). Equal volumes of 10 μM primer-F and primer-R were mixed with a slow annealing to obtain the *gRNA* oligo. For *gRNA* assembly, *gRNA* oligos of *P*_*INO2*_, *P*_*ERG7*_, and *LPP1* were introduced to the *Cas9* vector respectively, using Minerva Super Fusion Cloning Kit (Yuheng Biotech, Suzhou, China). Then, the vector of pCAS-*RNR2*p-*Cas9*-*CYC1*t was introduced into the engineered strain BY-II using LiAc/ssDNA method. A total of 1 μg of the *gRNA* expression plasmid and 1 μg of target fragment were co-transformed into BY-II, then cultivated on selective YPD medium containing 100 mg/L G418 sulfate (Sangon Biotech, Shanghai, China) at 30 °C for 2–3 d. Positive colonies were verified by sequencing.

### Yeast cultivation and metabolite extraction

Engineered strains were grown in YPD medium containing 100 mg/L G418 sulfate. To determine the performance of the engineered strains, positive colonies were cultivated in YPD medium for 72 h (30 °C, 220 rpm). Two mL fermented broth were centrifugated and cells were washed with distilled water at 12,000 rpm for 10 min. Next, cells were crushed by a high-throughput grinder (SCIENTZ-48, Ningbo Scientz Biotechnology Co., Ltd, Ningbo, China), followed by extraction with 0.6 mL methanol: acetone (1:1, v/v) 3 times.

### Chemical analysis

The fermentation broth was centrifugated and properly diluted. The concentrations of glucose and ethanol were detected using a biosensing analyzer (SBA-40C, Shandong Academy of Sciences, China). The quantification of DM-II and PPD were conducted using a SHIMADZU LC20A system (Shimadzu, Kyoto, Japan) equipped with LC-20ADXR liquid chromatograph and SIL-20AXR auto-sampler. Chromatographic separation of PPD was carried out at 30 °C on a Poroshell 120 EC-C18 column (4.6 × 250 mm, 4 μm, Agilent). DM-II and PPD were separated by using 10% water and 90% acetonitrile. The injection volume was 10 μL, and the flow rate was kept at 1.0 mL/min.

### Batch and fed-batch fermentation for PPD production

For batch fermentation, strain BY-V was inoculated into the YPD medium and cultivated at 30 °C on a rotary shaker at 220 rpm for 18 h. Then, the seed culture was added to 50 mL YPD medium in 250-mL flasks with a 2.0% inoculation and grown at 30 °C and 220 rpm for 72 h. The optical density at 600 nm (OD_600_) was measured using a Shimadzu UV-1900i spectrophotometer. Dry cell weight was calculated using the coefficient, 1 OD_600_ = 0.3296 g/L DCW.

For restricted glucose feeding, 0.4 mL glucose (or 40 g/L molasses) and 0.6 mL fed solution were added to the medium at 48, 60, 72, 84, 96, 108, 120, and 144 h. For the two-stage feeding, 10 g/L glucose/molasses and 0.6 mL fed solution (9 g/L KH_2_SO_4_, 5.12 g/L MgSO_4_·7H_2_O, 3.5 g/L K_2_SO_4_, 0.28 g/L Na_2_SO_4_, 1.5 g/L lysine, 12 mL vitamin solution, and 10 mL trace metal solution) were added at 24 and 36 h [54]. Ethanol was fed at intervals to maintain a concentration in the range of 1–5 g/L after 48 h. For restricted ethanol feeding, 0.2 mL ethanol (99.7%, v/v) and 0.6 mL fed solution were added to the medium at 48, 60, 72, 84, 96, 108, 120, and 144 h.

Fermentation by strain BY-V was conducted in a 5-L bioreactor (Sartorius Stedim Biotech, Gottingen, Germany) using synthetic medium (40 g/L molasses, 15 g/L (NH_4_)_2_SO_4_, 8 g/L KH_2_PO_4_, 1.5 g/L lysine, 5.65 g/L MgSO_4_, 0.72 g ZnSO_4_, 12 mL vitamin solution and 10 mL trace metal solution). 150 mL seed solution cultured at 30 °C and 220 rpm for 18 h was inoculated into 1.5 L synthetic medium. Fermentation was carried out at 30 °C and pH was controlled at 5.5 by aqueous ammonia. Dissolved O_2_ was maintained at 40% with an air flow rate higher than 1 L/min. Feeding rate was controlled at a range of 1–5 g/L.

### Statistical analysis

The experimental data were represented as mean ± standard deviation of biological triplicates. The statistical analyses were performed with GraphPad Prism software (San Diego California, USA) and Origin 9.6 (Origin Lab, Northampton, MA, USA).

## Supplementary Information

**Additional file 1: Table S1.** PPD production of engineering *Saccharomyces cerevisiae*. **Table S2.** Plasmids used in this study. **Table S3.** Primers used in this study. **Table S4.** Primers used for RT-PCR in this study. **Table S5.** *gRNA* target sequences used in this study. **Figure S1.** High-throughput screening of PPD-producing strains. **Figure S2.** RT-qPCR of PPD-producing strains. **Figure S3.** PPD production of strain BY-V with glucose/molasses feeding. **Figure S4.** Construction of *Cas9* expression plasmid. **Figure S5.** PPD production in a 5-L bioreactor. Supplementary Sequences.

## Data Availability

All data generated or analyzed during this study are included in this published article and additional file.
