# Supplementary material for: High-yield production of protopanaxadiol from sugarcane molasses by metabolically engineered Saccharomyces cerevisiae
Source: Microb Cell Fact. 2022 Nov 5;21:230. doi: 10.1186/s12934-022-01949-4 (PMC9636795; doi:10.1186/s12934-022-01949-4)
Supplement: Supplementary file 1 — Additional file 1: Table S1. PPD production of engineering Saccharomyces cerevisiae. Table S2. Plasmids used in this study. Table S3. Primers used in this study. Table S4. Primers used for RT-PCR in this study. Table S5. gRNA target sequences used in this study. Figure S1. High-throughput screening of PPD-producing strains. Figure S2. RT-qPCR of PPD-producing strains. Figure S3. PPD production of strain BY-V with glucose/molasses feeding. Figure S4. Construction of Cas9 expression plasmid. Figure S5. PPD production in a 5-L bioreactor. Supplementary Sequences. [file 12934_2022_1949_MOESM1_ESM.docx]

**Additional file**

**High-yield production of protopanaxadiol from sugarcane molasses by metabolically engineered *Saccharomyces cerevisiae***

Yuan Zhu^1,2^*^§^*, Jianxiu Li^2^*^§^*^*^, Longyun Peng^2^*^§^*, Lijun Meng^2^, Mengxue Diao^2^, Shuiyuan Jiang^3^, Jianbin Li ^1*^ and Nengzhong Xie^2*^

^1^ *College of Light Industry and Food Engineering, Guangxi University, 100 Daxue Road, Nanning, 530004, China;*

*^2^ State Key Laboratory of NonFood Biomass and Enzyme Technology, National Engineering Research Center for Non-Food Biorefinery, Guangxi Biomass Engineering Technology Research Center, Guangxi Key Laboratory of Bio-refinery, Guangxi Academy of Sciences, 98 Daling Road, Nanning, 530007, China.*

^3^ *Guangxi Institute of Botany, Guangxi Zhuangzu Autonomous Region and the Chinese Academy of Sciences, Guilin 541006, China.*

^*^Corresponding Author

(Jianbin Li) Email: lijb0771@sina.com. Phone: +86-139-7860-9908;

(Jianxiu Li) E-mail: jianxiuli@gxas.cn. Phone: +86-138-7715-2209;

(Nengzhong Xie) Email: xienengzhong@gxas.cn. Phone: +86-156-7619-2313.

^§^Y. Z., J. L. and L. P. contributed equally to this work.

**Table of Contents**

**Supplementary Tables**

**Table S1.** PPD production of engineering *Saccharomyces cerevisiae*.

**Table S2.** Plasmids used in this study.

**Table S3.** Primers used in this study.

**Table S4.** Primers used for RT-PCR in this study.

**Table S5.** *gRNA* target sequences used in this study.

**Supplementary Figures**

**Figure S1.** High-throughput screening of PPD-producing strains.

**Figure S2.** RT-qPCR of PPD-producing strains.

**Figure S3.** PPD production of strain BY-V with glucose/molasses feeding.

**Figure S4.** Construction of *Cas9* expression plasmid.

**Figure S5**. PPD production in a 5-L bioreactor.

**Supplementary Sequences**

**Supplementary Tables**

**Table S1.** PPD production of engineering *Saccharomyces cerevisiae.*

| **Strains**  **(Parent strains)** | **Related gene cassettes** | **Fermentation time (h)** | **PPD**  **(mg/g DCW)** | **PPD**  **(mg/L)** | **Fed-batch fermentation(g/L)** | **References** |
| --- | --- | --- | --- | --- | --- | --- |
| ZD-PPD-018  (BY4742) | *rDNA :: P_PGK1_-PgDDS*, *P_TDH3_-AtCPR1*, *P_TEF1_-PgPPDS*  *δDNA :: P_PGK1_-tHMG1*, *P_TDH3_-AtCPR1*, *P_TEF1_-SynPgPPDS, P_PGK1_-ERG20*, *P_TDH3_-ERG1*, *P_TEF1_-ERG9* | 144 | 13.11±0.96 | 148 | 1.19 (7.5 L) | [1] |
| GY-1  (BY4742) | *δDNA :: P_PGK1_-tHMG1*, *P_TEF1_-LYS2*, *rDNA :: P_PGK1_-GgbAS*, *P_TDH3_-ERG1*,*P_TEF1_-ERG9*  *Trp1 :: P_PGK1_-GgbAS*, *P_TDH3_-AtCPR1*, *P_TEF1_-MtOAS*  *His3 :: P_PGK1_-PgDDS*, *P_FBA1_-SynPgPPTS*, *P_TDH3_-AtCPR1*, *P_TEF1_-SynPPDS* | 168 | 1.4 | 17.2 | - | [2] |
| IN-B  (INVSc1) | *P_GAL10_-DS-GFP*, *P_GAL1_-ATR1*, *P_GAL1_-PPDS*, *P_GAL10_-ERG7*, *P_GAL1_-tHMG1* All plasmids were integrated into INVSc1 | 240 | 3.3 | 11.45±1.56 | - | [3] |
| W3  (W303-1a) | *MATa* {*leu2-3,112 trp1-1 can1-100 ura3-1 ade2-1 his3-11,15*}  *PPDS, ATR1,* and marker *URA3* expression cassettes were integrated into *δ* site of W2 | 144 | - | 155.3±+6.2 | 1.44 (5 L) | [4] |
| W3a-ssPy  (W303-1a) | *MATa* {*leu2-3,112 trp1-1 can1-100 ura3-1 ade2-1 his3-11,15*}  *PPDS, ATR1,* and marker *URA3* expression cassettes were integrated into *δ* site of W2  URA3 and His3 markers were deleted using a Cre-expressing plasmid pSH63  *PGK1p*-*SSD1*-*ADH1* cassette was inserted into his3 site of W3aHU  *PGK1p*-*YBP1* cassette was inserted into ura3 site of W3a-ss | 168 | - | - | 4.25 ± 0.18 (5 L) | [5] |
| WLT-MVA5  (W303-1a) | *δDNA :: P_ALD6_-DS*, *P_TDH3_-PPDS-ATR1*, *P_TEF1_-ERG1*  *rDNA :: P_TEF1_-tHMG1*, *P_ACS1_-ERG9*, *P_TEF2_-ERG20*, *P_TEF2_-ERG10*, *P_MLS1_-ERG13*, *P_ALD6_-ERG12*, *P_SSA1_- ERG8*  *HIS3 :: P_CIT2_-ERG19*, *P_IDP2_-IDI1*, *P_PGK1_-NCP1*  *LEU2 :: P_ACS1_-TetR*, *P_ERG7_ :: P _ERG7C_* , *HIS3 :: P_ADH2_-DS*, *P_TEF1_-ACS_seL641P_* | 144 | 66.55  (mg/g OD_600_) | 411.27 | 8.09 (5 L) | [6] |
| PPD08  (CEN.PK2-1D) | *LEU2 :: P_TEF1_-ERG20, HIS3 :: P_CCW12_-tHMG1*  *LPP1 :: P_GPD_-AtCPR1*, *DPP1 :: P_ADH2_-PgDS*  *YPL062w :: P_ADH2_-PgPPDS, ALD2 :: P_GPD_-ALD6* | 144 | 0.87 | 6.01 | - | [7] |
| ZW04BY-RS  (BY4742) | *δDNA :: P_HXT7_-tHMG1*, *P_TEF2_-synPgCPR1*,*P_TPII_-ERG1*, *P_GPM1_-ERG20*, *P_PGK1_-ERG9*, *P_TDH3_-synDDS*, *P_TEF1_-synPPDS*  *YPRCδ15 DNA :: P_ENO2_-ERG12*, *P_TEF2_-ERG13*, *P_TPI1_-ERG8*, *P_GPM1_-ERG19*,  *P_PGK1_-IDI*, *P_TDH3_-ERG10*, *P_TEF1_-tHMG1*  *rDNA :: P_TDH3_-synPPDS* | 96 | 41.1 | 529.0 ± 25.5 | 11.02 (10 L) | [8] |
| PPD-INO2  (CEN.PK2-1D) | *LEU2 :: P_TEF1_-ERG20*, *HIS3 :: P_CCW12_-tHMG1*  *LPP1 :: P_GPD_-AtCPR1, DPP1 :: P_ADH2_-PgDS*  *YPL062w :: P_ADH2_-PgPPDS*, *P_INO2_::P_PGK1_* | 144 | 0.98 | 12.1±1.0 | - | [9] |
| BY-V  (BY4742) | *delta17:: P_ADH1_-ERG20*, *P_HXT7_-ERG9*, *P_PGI_-ERG1*, *P_RPL8A_-PgDDS*, *P_ADH1_-PgPPDS-AtCPR1*, *P_TDH3_-tHMG1*  *delta15 :: P_ENO2_*-*ERG12*, *P_TEF2_-ERG13*, *P_GPM1_-MVD1*, *P_TPI1_-ERG8*, *P_GPD1_-IDI1*, *P_TDH3_-tHMG1*, *P_TEF1_-ERG10*  *P_INO2_ :: P_TEF1_*, *P_ERG7_ :: P_HXT1_*, *LPP1:: P_PGK1_-UPC2-1* | 72 | 78.13±0.38 | 562±1.65 | 15.88±1.65 (5 L) | This work |

**Table S2.** Plasmids used in this study.

Empty plasmids used to construct cassettes.

| **Name** | **Promoter1** | **Terminator1** | **Promoter2** | **Terminator2** |
| --- | --- | --- | --- | --- |
| p-T1 | *P_ADH1_* | *T_TPI1_* | *P_HXT7_* | *T_PGIT_* |
| p-T2 | *P_PGI1_* | *T_ADH1_* | *P_RPL8A_* | *T_CYC1_* |
| p-T3 | *P_TDH3_* | *T_PDC1_* | *P_PGK1_* | *T_TDH1_* |
| p-T4 | *P_CPS1_* | *T_ENO2_* | *P_TEF2_* | *T_IDP1_* |
| p-T5 | *P_GPM1_* | *T_HIS5_* | *P_TPI1_* | *T_PRM5_* |
| p-T6 | *P_GPD1_* | *T_RPM9_* | *P_TEF1_* | *T_SPG5_* |

Plasmids of gene expression cassettes.

| **Name** | **ORF1** | **ORF2** |
| --- | --- | --- |
| p-T1-*ERG20*-*ERG9* | *P_ADH1_-ERG20-T_TPI1_* | *P_HXT7_-ERG9-T_PGIT_* |
| p-T2-*ERG1*-*PgDDS* | *P_PGI1_-ERG1-T_ADH1_* | *P_RPL8A_-PgDDS-T_CYC1_* |
| p-T3*-tHMG1-PgPPDS-AtCPR1* | *P_TDH3_-tHMG1-T_PDC1_* | *P_PGK1_-PgPPDS-AtCPR1-T_TDH1_* |
| p-T4-*ERG12*-*ERG13* | *P_CPS1_-ERG12-T_ENO2_* | *P_TEF2_-ERG13-T_IDP1_* |
| p-T5-*MVD1*-*ERG8* | *P_GPM1_-MVD1-T_HIS5_* | *P_TPI1_-ERG8-T_PRM5_* |
| p-T6-*IDI1*-*ERG10* | *P_GPD1_-IDI1-T_RPM9_* | *P_TEF1_-ERG10-T_SPG5_* |

Other plasmids used in this study.

| **Name** | **Function** | **Resource** |
| --- | --- | --- |
| pUC57 | Protopanaxadiol expression Plasmid; Amp^r^ | Genscript |
| pYES3-CT | selection marker amplification; Amp^r^ | Invitrogen |
| p-RNR2p-*Cas9*-*CYC1*t | Guide *RNA* and *Cas9* expression plasmid | This study |
| pCAS-*Cas9*-G418 | Gene construction plasmid; Kan^r^, G418^r^ | This study |
| G4-*P_TDH3_* | *P_TDH3_* target fragment. Kan^r^, G418^r^ | This study |
| G4-*P_HXT1_* | *P_HXT1_* target fragment. Kan^r^, G418^r^ | This study |
| G4-*P_PGK1_*-*UPC2-1-T_ADH1_* | *P_PGK1_*-*UPC2-1-T_ADH1_* target fragment. Kan^r^, G418^r^ | This study |

**Table S3.** Primers used in this study.

| **Primers** | **Sequences (5'-3')** |
| --- | --- |
| U detal17-F | CGTCTCCCCCGGTCCGTTTG |
| U detal17-R | TCTGACTCCCCTGCTTTGACGTT |
| D detal17-F | GACAAAGCGCCAAGGAACTG |
| D detal17-R | TAGTCCGCGAGTTGGATAGC |
| U detal15-F | gaaatgtagattcattttgtagattcc |
| U detal15-R | tattcaccaattaatcacaagttggtaa |
| D detal15-F | aatataagaatccccaaaattgaatcggtat |
| D detal15-R | ataaagcagccgctaccaaacag |
| His-F | CTAGTACACTCTATATTTTTTTATGCCTC |
| His-R | CTACATAAGAACACCTTTGGTGGAG |
| TPI1 Ter-F | AGTCCCCTCTAAAATTGCCTCTATATAACAGTTGAAATTTGGATAAGAACA |
| TPI1 Ter-R | AAAGTCTACAAGAGATCTAAGTAAGATTAATATAATTATAT |
| ERG20-F | TTTATATAATTATATTAATCTTACTTAGATCTCTTGTAGACTTTATTCAAAAA |
| ERG20-R | catacaatcaactatctcatatacagATGGCATCCGAAAAAGAA |
| 1ADH1 Pro-F | ATTTCTTTTTCGGATGCCATctgtatatgagatagttgatt |
| 1ADH1 Pro-R | gcgtgctgccactcctaacatgtaggtggcggagg |
| 2HXT7 Pro-F | cctacatgttaggagtggcagcacgctaattcg |
| 2HXT7 Pro-R | AATTGCAACAATTTACCCATtttttgattaaaattaaaaaaa |
| ERG9-F | tttttaattttaatcaaaaaATGGGTAAATTGTTGCAATTGGC |
| ERG9-R | TTAAGAGCGATTTGTTCGATTTAAGCTCTATGCAAAGTATAAATATAATAAAAA |
| PGI Ter-F | CTTTGCATAGAGCTTAAATCGAACAAATCGCTCTTAAATA |
| PGI Ter-R | TATATTACAGTTCCTTGGCGCTTTGTCGGTATACTGGAGGCTTCATG |
| ADH1 Ter-F | TATCCAACTCGCGGACTATCGGCATGCCGGTAGAGGTG |
| ADH1 Ter-R | TCGGTGAATTGATTGGTTAAAGTTATAAAAAAAATAAGTG |
| ERG1-F | CACTTATTTTTTTTATAACTTTAACCAATCAATTCACCGAACA |
| ERG1-R | ctatttgtttcgtcccattgATGTCAGCCGTTAACGTT |
| PGI Pro-F | AACGTTAACGGCTGACATcaatgggacgaaacaaatagg |
| PGI Pro-R | tggcgtaatcatggtcacactacttctacacatcaacggtac |
| 4RPL8A Pro-F | ccgttgatgtgtagaagtagtgtgaccatgattacgccaagc |
| 4RPL8A Pro-R | CTTTCAACTTCCACATggagctcgatgtgataatag |
| PgDDS-F | ctattatcacatcgagctccATGTGGAAGTTGAAAGTTGC |
| PgDDS-R | tgacataactaattacatgaTCAGATCTTCAATTGCTGGT |
| CYCI Ter-F | GATCTGAtcatgtaattagttatgtca |
| CYCI Ter-R | GTTGGCGTCTACCGTCGTTgcaaattaaagccttcgagcg |
| FBA1 Ter-F | ACTAAGTCCGCggtaccGCTATCAAAAACGATAGATCGATTAGG |
| FBA1 Ter-R | AGATGTTTGGTAAACCTaagcttGTTAATTCAAATTAATTG |
| 46ATR1-F | TTGAATTAACaagcttAGGTTTACCAAACATCTCTCAAAT |
| 46ATR1-R | CTACTTCTTCAGGTTCAGGTTGGAAGAAAACTACTGCTGA |
| PgPPDS-F | ACCTGAACCTGAAGAAGTAGAACCGTTATGTGGATGCAAGTGAATTGG |
| PgPPDS-R | aatcaactatctcatatacaATTCATGGTCCTGTTCTTCTCTCT |
| ADH1 Pro-F | GAAGAACAGGACCATGAATtgtatatgagatagttgatt |
| ADH1 Pro-R | AACATTCAACGCTAGTATacatgtaggtggcggagggg |
| TDH3 Pro-F | cctccgccacctacatgtATACTAGCGTTGAATGTTAGCG |
| TDH3 Pro-R | GTTTTAACCAATTGGTCCATcTTTGTTTGTTTATGTGTGTTTATTCG |
| tHMG1-F | AAACACACATAAACAAACAAAgATGGACCAATTGGTTAAAAC |
| tHMG1-R | TAATAATTAGAGATTAAATCGCCGATTTAAGATTTAATACAAGTAACAGA |
| PDC1 Ter-F | TGTATTAAATCTTAAATCGGCGATTTAATCTCTAATTATTA |
| PDC1 Ter-R | CTTAGCCTCTGTATCGTCTGGgagctcTGTTCCTTAATCAAGGATACC |
| Leu-F | ttgtgattaattggtgaataTTAACTGTGGGAATACTCAGGTATCG |
| Leu-R | TGTCAAATCAAGTGTCAAATTCTACCCTATGAACATATTCCA |
| CPS1 Ter-F | GTTGGAACCTggtaccATTTGACACTTGATTTGACACTTCTTT |
| CPS1 Ter-R | acaaattgacgatctattattgccaggaaacacgaatttaccatggacttcataaGCGCAATGATTGAATAGTCAA |
| ERG12-F | TTGACTATTCAATCATTGCGCttatgaagtccatggtaaattcgtgt |
| ERG12-R | ATACTATAACATACAATAATAatgtcattaccgttcttaacttctgc |
| ENO2 Pro-F | gttaagaacggtaatgacatTATTATTGTATGTTATAGTATTAGTTGCTTGGTG |
| ENO2 Pro-R | TATGTAAGTATACGGCCCCATCGACGCTGCGGGTATAGAA |
| TEF2 Pro-F | CTATACCCGCAGCGTCGATGGGGCCGTATACTTACATATAGTAGA |
| TEF2 Pro-R | gcggcctaagtcttcctttaataccacaccaacaaagtttagttgagagtttcatGTTTAGTTAATTATAGTTCGTTGACCGTAT |
| ERG13-F | CGAACTATAATTAACTAAACatgaaactctcaactaaactttgt |
| ERG13-R | TGGGCTACGTAAATTCGAttattttttaacatcgtaagatcttctaaatttg |
| IDP1 Ter-F | cttacgatgttaaaaaataaTCGAATTTACGTAGCCCAATCTAC |
| IDP1 Ter-R | GCTAATCTGACTGGTTGGCTGGATGGTAATGATCCGAACTTGGG |
| HIS5 Ter-F | TTGGAACCTggtaccGTAACAATATCATGAGACCTTTTATAGAAGTG |
| HIS5 Ter-R | ctggtctaccaaaggaataaATAGATTAATTTAAACAGTATATGTACAGTTTTATATATAT |
| MVD1-F | GTTTAAATTAATCTATttattcctttggtagaccagtctttg |
| MVD1-R | CCAAACAAACACACATATTACAATAatgaccgtttacacagcatc |
| GMP1 Pro-F | ttgcgatgttgacgggtgcggtaacggatgctgtgtaaacggtcatTATTGTAATATGTGTGTTTGTTTGGATT |
| GMP1 Pro-R | CCAACCTGATGGGTTCCTAGATATAAGTCGTGCAATGTATGA |
| TPI1 Pro-F | AAGTCATACATTGCACGACTTATATCTAGGAACCCATCAGGTTGG |
| TPI1 Pro-R | aaggctctcaactctgacatTTTTAGTTTATGTATGTGTTTTTTGTAGTTATAGA |
| ERG8-F | CACATACATAAACTAAAAatgtcagagttgagagccttcagtg |
| ERG8-R | AAAAAAATATTGCAAAATATCATAAAAGTTTttatttatcaagataagtttccggatctttttcttt |
| PRM5 Ter-F | ggctgactggggtgttaggaaagaaaaagatccggaaacttatcttgataaataaAAACTTTTATGATATTTTGCAATATTTTTTT |
| PRM5 Ter-R | GCTAATCTGACTGGTTGGCTGATAGAACCCAAAAAGAGAGACTAAACAA |
| PRM9 Ter-F | AGGTTCCAACTGCTCTTACTGT |
| PRM9 Ter-R | GTTGGAACCTggtaccATTTTCAACATCGTATTT |
| IDI1-F | GTGTCTCCCGTCTTCTGTttatagcattctatgaatttgcctgtc |
| IDI1-R | CAAATATTGATAATATAAAGatgactgccgacaacaatagtatgc |
| GPD1 Pro-F | ttgttgtcggcagtcatCTTTATATTATCAATATTTGTGTTTGTGGA |
| GPD1 Pro-R | agaaacattttgaagctatgGCAAGGTGACAGCGGTCC |
| TEF1 Pro-F | AGACGGGGACCGCTGTCACCTTGCcatagcttcaaaatgtttctactcc |
| TEF1 Pro-R | tgtaaacgttctgagacatcttagattagattgctatgc |
| ERG10-F | gcatagcaatctaatctaagatgtctcagaacgtttacattgtatcg |
| ERG10-R | GCGATGAAACAACGTCTTTGtcatatcttttcaatgacaatagaggaa |
| SPG5 Ter-F | cattgaaaagatatgaCAAAGACGTTGTTTCATCGCG |
| SPG5 Ter-R | GCTAATCTGACTGGTTGGCTGGCTTATTTTCTGCCGAATTTTC |
| UINO2-F | GCGTGTTTATGCTTAAATGCGGTCACTCCTGTAAGCTCGTCC |
| UINO2-R | TTCCCAGTTGCTTGTTGCATGTCAAGGATATGAGTTTATGGT |
| DINO2-F | CATAAACTCATATCCTTGACATGCAACAAGCAACTGGGAAC |
| DINO2-R | TGGCCATAGAAAAATTCTGTTTAATTCGGTATACCTATGCTGCTT |
| URA3-F | CATAAACTCATATCCTTGACTTCAATTCATCATTTTTTTTTTATTCTTT |
| URA3-R | CATACCCCTCATTTCCACGGGGGTAATAACTGATATAATTAAATTGAAGCT |
| HXT7-Pro-F | AATTATATCAGTTATTACCCCCGTGGAAATGAGGGGTATG |
| HXT7-Pro-R | TTCCCAGTTGCTTGTTGCATATTTTTTGATTAAAATTAAAAAAACT |
| PGK1-Pro-F | AATTATATCAGTTATTACCCACGCACAGATATTATAACATCTGCAC |
| PGK1-Pro-R | TTCCCAGTTGCTTGTTGCATTGTTTTATATTTGTTGTAAAAAGTAGATAATTACT |
| TEF1-Pro-F | AATTATATCAGTTATTACCCAGTGATCCCCCACACACCATAG |
| TEF1-Pro-R | TTCCCAGTTGCTTGTTGCATTTTGTAATTAAAACTTAGATTAGATTGCT |
| TDH3-Pro-F | AATTATATCAGTTATTACCCATACTAGCGTTGAATGTTAGCGTCA |
| TDH3-Pro-R | TTCCCAGTTGCTTGTTGCATTTTGTTTGTTTATGTGTGTTTATTCGA |
| UERG7-F | AGAtaataccccttgaggagaatgt |
| UERG7-R | ttcaggaggggggaattatattccagatgagaTCGACTTTATCGAGGAACACTAATACA |
| DERG7-F | taatcgtcaactagttgatatacgtaaaatcATGACAGAATTTTATTCTGACACAATCG |
| DERG7-R | GGTCCTTGGGTAGACCCAATA |
| HXT1 Pro-F | TCAAGAGTTGTATTAGTGTTCCTCGATAAAGTCGAtctcatctggaatataattccccc |
| HXT1 Pro-R | GACCGATTGTGTCAGAATAAAATTCTGTCATgattttacgtatatcaactagttgacga |
| ADH1 Ter-F | gtgtttatgcttaaatgcTCGGCATGCCGGTAGAGG |
| ADH1 Ter-R | AGTTATAAAAAAAATAAGTGTATACAAATTTTAAAG |
| *UPC2-1*-F | CACTTATTTTTTTTATAACTTCATAACGAAAAATCAGAGAAA |
| *UPC2-1*-R | TTTACAACAAATATAAAACAATGAGCGAAGTCGGTATACAGAATC |
| PGK1-TEF1-F | TGTATACCGACTTCGCTCATTGTTTTATATTTGTTGTAAAAAGTAGATAATTACT |
| PGK1-TEF1-R | GTTTTAACCAATTGGTCCATTTTGTAATTAAAACTTAGATTAGATTGCTATG |
| ROX1-tHMG1-F | ATCTAAGTTTTAATTACAAAATGGACCAATTGGTTAAAACTGAA |
| ROX1-tHMG1-R | TGACATAACTAATTACATGATTAAGATTTAATACAAGTAACAGAACCATCTT |
| CYC1 Ter-F | TCATGTAATTAGTTATGTCACGCTTACA |
| CYC1 Ter-R | catagaaaaattctgtGCAAATTAAAGCCTTCGAGCG |
| ULPP1-F | AAAGGGGCAGAAGCAAGATT |
| ULPP1-R | CACCTCTACCGGCATGCCGATAACACTTACAGAGTCCTATCAGGAAAGA |
| PUPC2-1-F | CGGCTTTTATTCTTTCCTGATAGGACTCTGTAAGTGTTATCGGCATGCCGGTAGAGGTG |
| PUPC2-1-R | CGCCTAAGGAAACTCGTCATATTCTACCAAGGACGCACAGATATTATAACATCTGCACA |
| DLPP1-F | TGCCTATTGTGCAGATGTTATAATATCTGTGCGTCCTTGGTAGAATATGACGAGTTTCC |
| DLPP1-R | CCAATCATGGTTTCATGGTCACTG |
| UROX1-F | ATCGGCCGCGTGGAACTACC |
| UROX1-R | AGGAGTAGAAACATTTTGAAGCTATGGTGTGTGGGGGATCACTTGTTGATTGTCTAACTGCGTTCTTTTGT |
| PtHMG1-F | CTTCTTCACACAAAAGAACGCAGTTAGACAATCAACAAGTGATCCCCCACACACCATAG |
| PtHMG1-R | TAATATAACGGAAAGAAGAAATGGAAAAAAAAAAGCAAATTAAAGCCTTCGAGCGTCCC |
| DROX1-F | GAAGGTTTTGGGACGCTCGAAGGCTTTAATTTGCTTTTTTTTTTCCATTTCTTCTTTCCGT |
| DROX1-R | CTGTTTAACAAAGCTTAACATTGTCAA |

**Table S4.** Primers used for RT-PCR in this study.

| **Target** | **Sequence（5ʹ-3ʹ）** |
| --- | --- |
| q-ARG4-F | ATGCCTCAGAAGAAGAATG |
| q-ARG4-R | ATGGAGTGCTCTACAGTT |
| q-ERG8 | GATGATTGCCAGACCTTAA |
| q-ERG8 | CAGCCATTGAACCTTAGAA |
| q-ERG13 | AGTCTGTCAAGTCTGTCT |
| q-ERG13 | ACTACAATGGCGTCTCTA |
| q-IDI1-F | GGACGATAATGCTATTGGT |
| q-IDI1-R | CAGTGGCTCTTTGTTGTA |
| q-ERG20 | GGTTGACCTCTTCTAGTA |
| q-ERG20 | TCTGTTGTTGATACTTATGCTA |
| q-PgPPDS-F | GTTGTTGACCAGAGAAGT |
| q-PgPPDS-R | AGAGGCAATATCAGATTCG |
| q-CPR1-F | GTTGCCAAGTAATCCATCTA |
| q-CPR1-R | CTTCACCATCTTCCTTCAA |
| q-tHMG1-F | TGTCTTCTGCTCAATCTTC |
| q-tHMG1-R | GCTTCCAATTCTTCCAATG |

**Table S5.** *gRNA* target sequences used in this study.

| **Target** | **Sequence（5ʹ-3ʹ）** |
| --- | --- |
| *ERG7p* | CTAGACCGTGTTATAGGCGC |
| *LPP1* | CACTTGTAGTATATCTAACT |
| *INO2p* | TAAGTACATGCGTATAGGTA |

**Supplementary Figures**


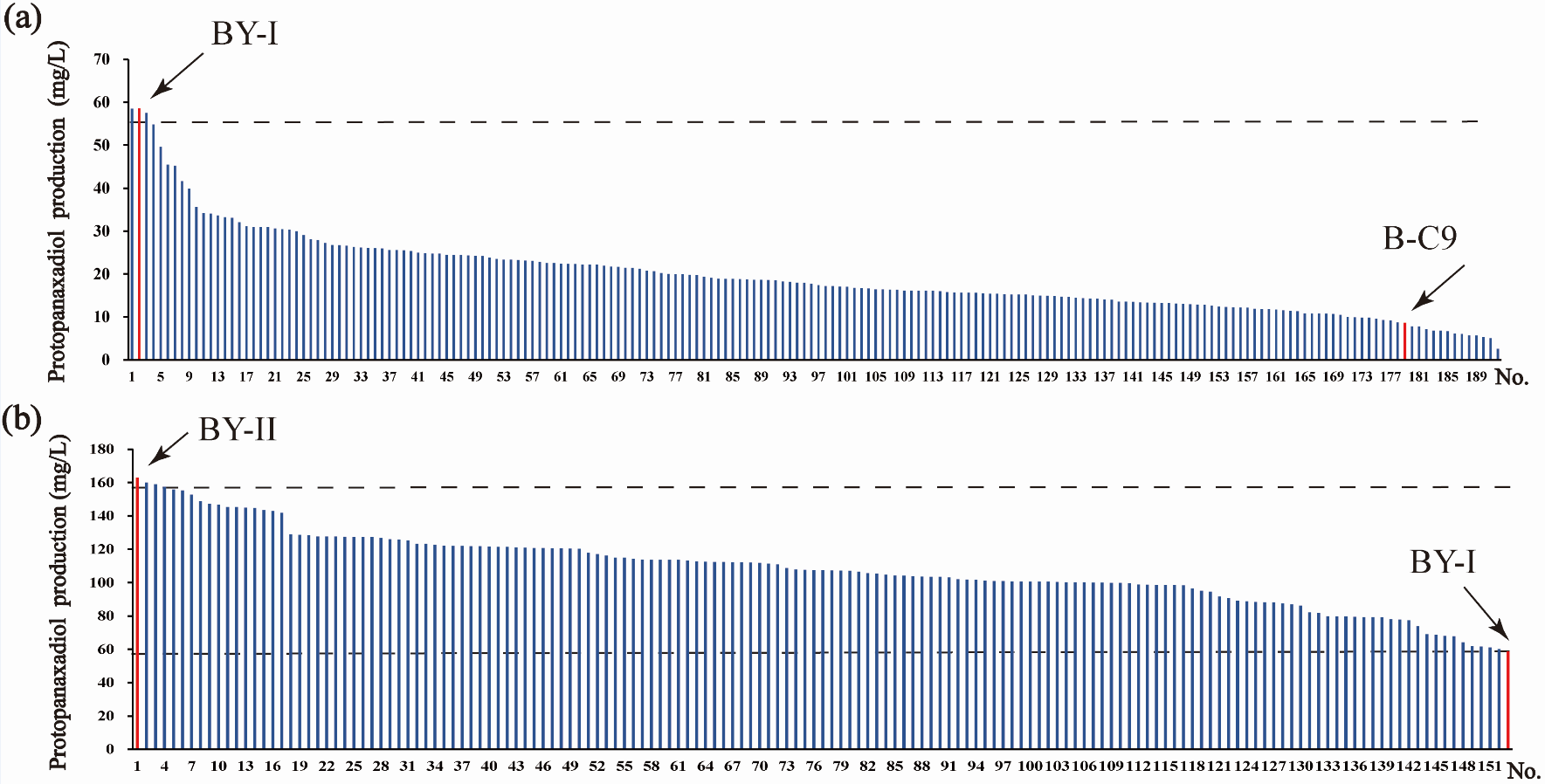


**Figure S1.** High-throughput screening of PPD-producing strains. (a) The first cassettes were integrated into the *delta17* multi-copy site of BY4742, red bars represent the PPD production of BY-I and B-C9. (b) The second cassettes were integrated into the *delta15* multi-copy site of BY-I, the red bar represents the PPD production of BY-II.


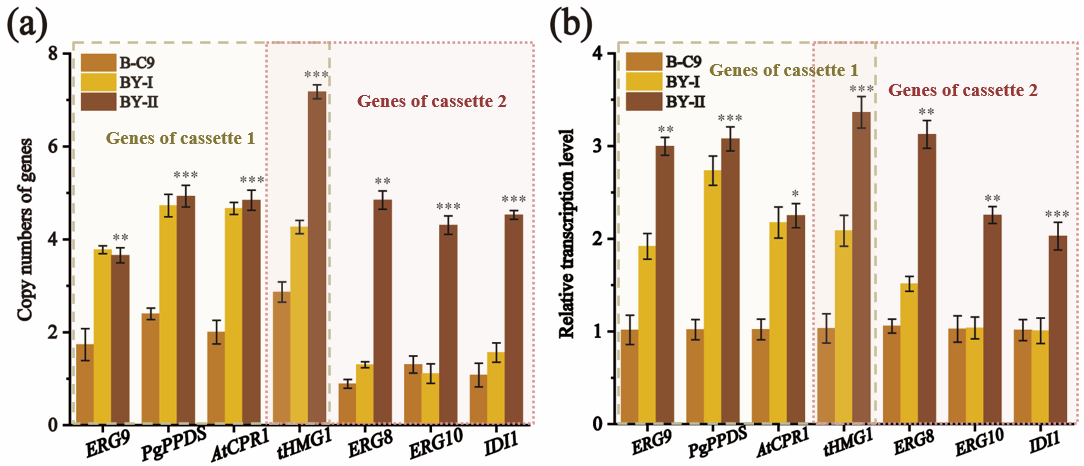


**Figure S2.** RT-qPCR of PPD-producing strains. (a) The copy numbers of genes in B-C9, BY-I and BY-II. (b) The relative transcription levels in B-C9, BY-I and BY-II. Two heterologous genes (*PgPPDS* and *AtCPR1*) and two endogenous genes (*tHMG1* and *ERG9*) of the first cassette and four endogenous genes (*tHMG1, ERG8, ERG10 and IDI1*) of the second cassette were detected using RT-qPCR. *ARG4* gene was used as a reference gene. Data are presented as the means of three replicates. ***P < 0.001, **P < 0.01, *P < 0.05.


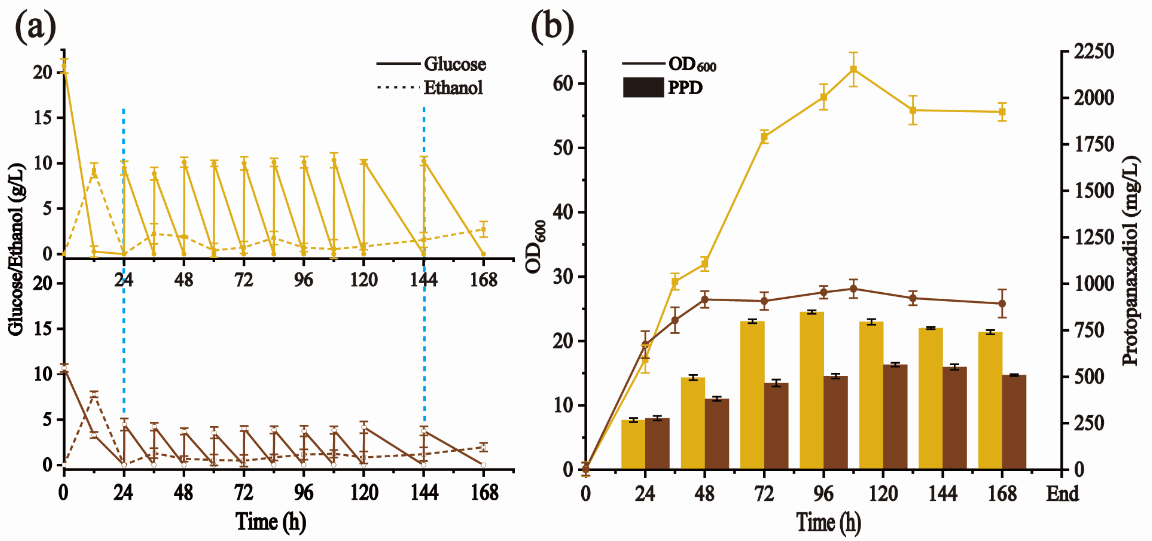


**Figure S3.** PPD production of chassis BY-V with glucose/molasses feeding. (a, b) Glucose/ethanol consumption, cell growth and PPD production of BY-V by restricted glucose/molasses feeding. The yellow curves and bars represent fermentation in glucose, and brown curves and bars represent fermentation in molasses. Data are presented as the means of three replicates, and bars represent the standard deviations.


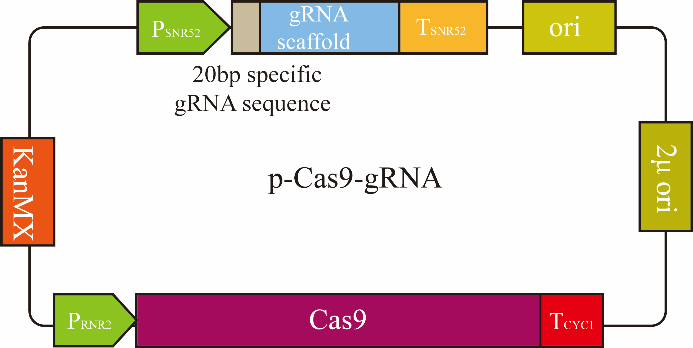


**Figure S4.** Construction of *Cas9* expression plasmid.


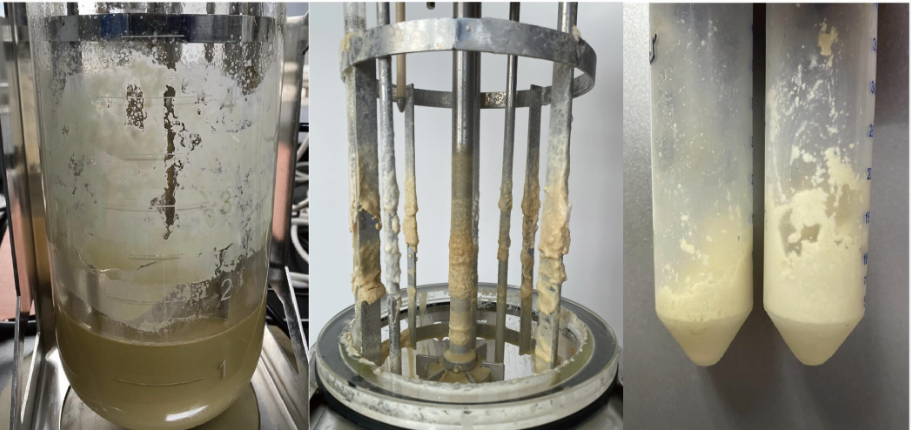


**Figure S5** PPD production in a 5-L bioreactor. Most of PPD was secreted to extracellular space. And they existed in medium as precipitation adhered to the surface of the inner tank wall and stainless pipe as yellow sediment.

**Supplementary Sequences**

> *ERG7p*

CGTTTTGTATACCTCTATTATGTAGTGCAAGAAAATCTGCTGCTATTCGTGATTACTGTTACAACCTAACGGTTTAAATGAAACCTGGTTCTGAAGGGTCATTTTATAACTTCAAGTTCCCTTAGCCTTTCGATTCATTTTGATTATGCCATTTCTAGACCGTGTTATAGGCGCTGGCGTTTAATTTGGTGTAGCTTGGTTTAGTCAAGAGTTGTATTAGTGTTCCTCGATAAAGTCGATGTTTCCGGATATTGTGTTAAAATTTCAAGTATGCTACTAATGGGGTAAAGTTGCATGATTAGCAGAGACATATGGCTTGTTATGGTTCGGCTTCCTCATTTTTCATGCTTAGTTTTTGTCCATCTCATTGTACATTTCTGAATCCTAATGCATGACTCCCTAACATTACTATTAAATTCTCAATAGTGAAGAATAAGCAAAATGGGAACCATGATAATTTCTAGCTTTCTCTCCACCCCTATTTTAATTTGCAATCATATATAGTACTTTCAATAGCATCTTTTCTAGATTTGATATCTGCGGAAACTTTTCGTTTATAATCGTTTAGGTGAAAAGTTTTTATATCGGTTATTTACAGATATACATTTTCTCAAAAAAAAAAAAATATAATACATGCCCTCAGCTTTTAATAAAGCCATTGAGCACAAGCCTCTCCAGTAATGTACTGCTGTGCCCAATAACCTTACCAATAATCGTCGCCCACAAAGAAAGTACAAAACAG

> *INO2p*

CATTTAGCAGCGCCAGCGCCCTTCTAAGCTCTTCCATACCTATACGCATGTACTTAAGCTGACTCTATCGAAAAATGACGTACGCACAAATGCACATGTATACATACGCAATCCATCCTTTATATAAGCTGAGAAACAAGCAGAGGAACCTGCTTCAGTTGGGCCATCCCTTCGATGCTATATACTTCTAATCTTAAACTCTTCACTAAGACTTCGCAGGCACCGCTTCAAGGATGTCTCAGGTCGCGAGATTACTACAACAACTTAAAACAACCCTGATGCAACTGCAGCGTGCTGTTTTTCAACAGTACAAGAAAAAGTTCCATATTGTGCCATTGCCGATGCCATTGCAAAGGAATTTGTTCTCAGTCGCATCTCTCCTTTACTGCAGATCTGAGTTACTTTGAATCCTTGATAATATGGATCGAGTGCCTCTTCCATTATTTCTATTATTATAATCTCAACGCTTAATATGAATTAACCAACCACACTCAAACCAAATCTACGCCAGCTACAAATGGTAGGAGAATGAAGACACACCAGGACTCAAACAGGATTTCCCAACAATGTCTTTCAACATGCTGCTTGCCGGTCACTACTGCACTTCCTGCGCCTTTTCCGTTGAATCACCCTCCATAGTGCGGCAGATGGAACCAACCTAACCTTTTTTCAGCAGGAGGTAAAAAATTCGCCGGTTTTCTATCTCCCTCCGTCATCGGCAGGGCGTTGACACCTGTCCAAGTACAACACACATGTGAATTTTTTTCAGTGATTAAAGGCTCATCTCATCATATCTTATTGTAATCAGTATTTGGCGTCATAAGCAATTCGAAATTGGTTCGGTTCCATCTCGTTGACGTGCAATAAATAAATACATGGAACAGCAAAGGAGAAA

> *UPC2.1*

ATGAGCGAAGTCGGTATACAGAATCACAAGAAAGCGGTGACAAAACCCAGAAGAAGAGAAAAAGTCATCGAGCTAATTGAAGTGGACGGCAAAAAGGTGAGTACGACTTCAACCGGTAAACGTAAATTCCATAACAAATCAAAGAATGGGTGCGATAACTGTAAAAGAAGAAGAGTTAAGTGTGATGAAGGGAAGCCAGCCTGTAGGAAGTGCACAAATATGAAGTTGGAATGTCAGTATACACCAATCCATTTAAGGAAAGGTAGAGGAGCAACAGTAGTGAAGTATGTCACGAGAAAGGCAGACGGTAGCGTGGAGTCTGATTCATCGGTAGATTTACCTCCTACGATCAAGAAGGAGCAGACACCGTTCAATGATATCCAATCAGCGGTAAAAGCTTCAGGCTCATCCAATGATTCCTTTCCATCAAGCGCCTCTACAACTAAGAGTGAGAGCGAGGAAAAGTCATCGGCCCCTATAGAGGACAAAAACAATATGACTCCTCTAAGTATGGGCCTCCAGGGTACCATCAATAAGAAAGATATGATGAATAACTTTTTCTCTCAAAATGGCACTATTGGTTTTGGTTCTCCTGAAAGATTGAATTCAGGTATCGATGGCTTACTATTACCGCCATTGCCTTCTGGAAATATGGGTGCGTTCCAACTTCAGCAACAGCAGCAAGTGCAGCAGCAATCTCAACCACAGACCCAAGCGCAGCAAGCAAGTGGAACTCCAAACGAGAGATATGGTTCATTCGATCTTGCGGGTAGTCCTGCATTGCAATCCACGGGAATGAGCTTATCAAATAGTCTAAGCGGGATGTTACTATGTAACAGGATTCCTTCCGGCCAAAACTACACTCAACAACAATTACAATATCAATTACACCAGCAGCTGCAATTGCAACAGCATCAGCAAGTTCAGCTGCAGCAGTATCAACAATTACGTCAGGAACAACACCAACAAGTTCAGCAACAACAACAGGAACAACTCCAGCAATACCAACAACATTTTTTGCAACAGCAGCAACAAGTACTGCTTCAGCAAGAGCAACAACCTAACGATGAGGAAGGTGGCGTTCAGGAAGAAAACAGCAAAAAGGTAAAGGAAGGGCCTTTACAATCACAAACAAGCGAAACTACTTTAAACAGCGATGCTGCTACATTACAAGCTGATGCATTATCTCAGTTAAGTAAGATGGGGCTAAGCCTAAAGTCGTTAAGTACCTTTCCAACAGCTGGTATTGGTGGTGTTTCCTATGACTTTCAGGAACTGTTAGGTATTAAGTTTCCAATAAATAACGGCAATTCAAGAGCTACTAAGGCCAGCAACGCAGAGGAAGCTTTGGCCAATATGCAAGAGCATCATGAACGTGCAGCTGCTTCTGTAAAGGAGAATGATGGTCAGCTCTCTGATACGAAGAGTCCAGCGCCATCGAATAACGCCCAAGGGGGAAGTGCTAGTATTATGGAACCTCAGGCGGCTGATGCGGTTTCGACAATGGCGCCTATATCAATGATTGAAAGAAACATGAACAGAAACAGCAACATTTCTCCATCAACGCCCTCTGCAGTGTTGAATGATAGGCAAGAGATGCAAGATTCTATAAGTTCTCTAGGAAATCTGACAAAAGCAGCCTTGGAGAACAACGAACCAACGATAAGTTTACAAACATCACAGACAGAGAATGAAGACGATGCATCGCGGCAAGACATGACCTCAAAAATTAATAACGAAGCTGACCGAAGTTCTGTTTCTGCTGGTACCAGTAACATCGCTAAGCTTTTAGATCTTTCTACCAAAGGCAATCTGAACCTGATAGACATGAAACTGTTTCATCATTATTGCACAAAGGTCTGGCCTACGATTACAGCGGCCAAAGTTTCTGGGCCTGAAATATGGAGGGACTACATACCGGAGTTAGCATTTGACTATCCATTTTTAATGCACGCTTTGTTGGCATTCAGTGCCACCCATCTTTCGAGGACTGAAACTGGACTGGAGCAATACGTTTCATCTCACCGCCTAGACGCTCTGAGATTATTAAGAGAAGCTGTTTTAGAAATATCTGAGAATAACACCGATGCGCTAGTTGCCAGCGCCCTGATACTAATCATGGACTCGTTAGCAAATGCTAGTGGTAACGGCACTGTAGGAAACCAAAGTTTGAATAGCATGTCACCAAGCGCTTGGATCTTTCATGTCAAAGGTGCTGCAACAATTTTAACCGCTGTGTGGCCTTTGAGTGAAAGATCTAAATTTCATAACATTATATCTGTTGATCTTAGCGATTTAGGCGATGTCATTAACCCTGATGTTGGAACAATTACTGAATTGGTATGTTTTGATGAAAGTATTGCCGATTTGTATCCTGTCGGCTTAGATTCGCCATATTTGATAACACTAGCTTATTTAGATAAATTGCACCGTGAAAAAAACCAGGGTGATTTTATTCTGCGGGTATTTACATTTCCAGCATTGCTAGACAAGACATTCCTGGCATTACTGATGACAGGTGATTTAGGTGCAATGAGAATTATGAGATCATATTATAAACTACTTCGAGGATTTGCCACAGAGGTCAAGGATAAAGTCTGGTTTCTCGAAGGAGTCACGCAGGTGCTGCCTCAAGATGTTGACGAATACAGTGGAGGTGGTGATATGCATATGATGCTAGATTTCCTCGGTGGCGGATTACCATCGATGACAACAACAAATTTCTCTGATTTTTCGTTATGA

> *PgDDS*

ATGTGGAAGTTGAAAGTTGCCCAAGGTAATGATCCATACTTGTACTCTACCAACAATTTCGTCGGTAGACAATACTGGGAATTTCAACCAGATGCTGGTACTCCAGAGGAACGTGAAGAGGTTGAAAATGCTAGAAAGGATTACGTCAACAACAAAAAGTTGCATGGTGTTCATCCATGCTCCGATATGTTGATGAGAAGGCAATTGATCAAAGAGTCCGGTATCGATTTGTTGTCTATTCCACCAGTTAGATTGGACGAAAACGAACAAGTTAACTACGATGCTGTTACTACCGCTGTTAAGAAAGCTTTGAGATTGAACAGAGCTATTCAAGCCCATGATGGTCATTGGCCAGCTGAAAATGCAGGTTCTTTGTTGTATACACCACCATTGATTATCGCCTTGTACATCTCTGGTACTATCGATACCATTTTGACCAAGCAGCACAAGAAAGAATTGATCAGATTCGTCTACAACCACCAGAATGAAGATGGTGGTTGGGGTTCTTATATTGAAGGTCATTCTACCATGATCGGCTCCGTTTTGTCTTTTGTCATGTTGAGATTACTAGGTGAAGGTTTGGCTGAATCTGATGATGGTAATGGTGCTGTTGAAAGGGGTAGAAAATGGATTTTGGATCATGGTGGTGCTGCTTCTATTCCATCTTGGGGTAAAACTTATTTGGCCGTTTTGGGTGTTTATGAATGGGAAGGTTGTAATCCATTGCCACCTGAATTTTGGTTGTTCCCATCATCTTTTCCATTCCATCCAGCTAAAATGTGGATCTACTGTAGATGTACTTACATGCCCATGTCTTACTTGTACGGTAAAAGATACCATGGTCCAATCACCGATTTGGTCTTGTCTTTGAGACAAGAGATCTACAACATTCCATACGAACAGATTAAGTGGAACCAGCAAAGACATAACTGTTGCAAAGAGGACTTGTACTACCCACATTCTTTGGTTCAAGATTTGGTTTGGGATGGCTTGCATTACTTTTCTGAACCATTTTTGAAGAGGTGGCCCTTCAACAAATTGAGAAAAAGAGGTCTTAAGAGGGTCGTCGAATTGATGAGATATGGTGCTACTGAAACCAGATTCATTACTACTGGTAACGGTGAAAAGGCCTTGCAAATTATGAGTTGGTGGGCTGAAGATCCTAATGGTGATGAATTCAAACATCACTTGGCCAGAATTCCAGATTTCTTGTGGATTGCCGAAGATGGTATGACCGTTCAATCTTTTGGTTCTCAATTGTGGGATTGCATTTTGGCTACCCAAGCTATTATTGCTACCAACATGGTTGAAGAATACGGCGATTCTTTGAAGAAGGCTCACTTCTTCATCAAAGAGAGCCAGATCAAAGAAAACCCAAGAGGTGATTTCTTGAAGATGTGTAGACAATTCACAAAAGGTGCTTGGACCTTCTCTGATCAAGATCATGGTTGTGTTGTTTCTGATTGCACTGCTGAAGCTTTGAAATGCTTGTTGTTGCTATCTCAAATGCCACAAGATATCGTTGGTGAAAAGCCAAAGGTTGAAAGATTATACGAAGCCGTTAACGTCTTGTTGTACTTGCAATCTAGAGTTTCAGGTGGTTTTGCTGTTTGGGAACCACCAGTTCCAAAGCCATATTTGGAAATGTTGAACCCATCCGAAATTTTCGCCGATATTGTCGTTGAACGTGAACATATTGAATGTACCGCCTCTGTTATTAAGGGTTTGATGGCTTTCAAGTGCTTGCATCCAGGTCATAGACAAAAAGAAATCGAAGATTCTGTTGCCAAGGCCATCAGATACTTGGAAAGAAATCAAATGCCTGACGGTTCTTGGTATGGTTTTTGGGGTATTTGTTTCTTGTACGGCACTTTCTTTACCTTGTCAGGTTTTGCTTCTGCTGGTAGAACTTACGATAATTCCGAAGCTGTTAGAAAAGGCGTCAAGTTCTTCTTGTCTACTCAAAACGAAGAAGGTGGCTGGGGAGAATCTTTGGAATCTTGTCCATCTGAAAAGTTCACTCCATTGAAGGGTAACAGAACTAACTTGGTACAAACTTCTTGGGCTATGTTGGGTTTAATGTTTGGTGGTCAAGCTGAAAGAGATCCAACTCCATTGCATAGAGCTGCTAAGTTGTTGATTAACGCCCAAATGGATAACGGTGATTTCCCACAACAAGAAATTACCGGTGTTTACTGCAAGAACTCCATGTTGCATTATGCTGAGTACAGAAACATTTTCCCATTGTGGGCTTTGGGTGAGTATAGAAAGAGAGTTTGGTTGCCAAAACACCAGCAATTGAAGATCTGA

> *PgPPDS*

GTTATGTGGATGCAAGTGAATTGGCAAACCATGAGCAAATCTAGGAATTGGGTCCGTCAGAATCTTTTCGTTGGGAATCAATTTTTCCCACCTGAAGTTAGTGACGACATTATGCATGAAGATCAAGATGACCAATCTGGCATATTCAATACCTGGACACATTCTTGGACCACCACCAAATGGAGTAAAAGTGTATGGAGCTGGACCATTACCTTCAAATCTAGTTGGATCGAACTTTTCTGGAGATGGAAAGTAAGTAGGGTTCTTATGAGTATCATGTGGGATCAAATGCATCTTCCAACCTTTTGGAATCAAGTAACCGGCGTAAGTGAAATCAGTAATAGCTTCTCTGAAAGTACCAACACCTGGTGGAATAATTCTCAAGACTTCTTGAGCAACGTTCCAAGAGTACTTCATCTTCCTCAAATCTTCCCAATTCAACAACTCTTTTGGGTGCTTAGAGTTAGCAATTTCGACCTGTTCTTTCAAGACCTGGTTGTAAACATCTGGGAATTCAGCCAAGTAGTTCAAGACGAAGGTAATAGTACCGTTCAAAGTAGTGTAACCACCTTGCATCAAACCGATCAAATGAGAGGCAATATCAGATTCGGACAAGAATTGACCATCTTGATTGGCAGTCAACAACAAGTGAGACAACAAATCTTGTGGTTGAGAAGCTTGCTTGTTTTCCAACAAGTCAACCTTTCTTTGCTTGATAACGGCTTCAACTTCTCTGGTCAACAACTTAACAGTCTTGATAGCTCTGTTCATAGCAGTACCTGGAATATTGATTGGAACGGCCAACAAACCAGCTTCAATGTTTTGAATGGAAGAACCCAACCTGGTGACTTGTTCTTCATCATCAATAGACATGAACACCCTGCAAGACATGGTAACAGTGTACTTTTTGACGGTGTTGTGAACGTTGATCTGTTGTTGTCTATTCCAATCGGTTTCCAAGAATTGCTTCATGACTCTATCCATCAAACCAACGTATTTCTTCATGCCGTCAACTTTCAACAAGAACATCATCTTACCCCTGACCTTAGAGAAGTTATCAGCATTAGATTCACCGTGAGATCTTGGGAACATCTTTTCAACAGAAGATGGGAACCAAACTTGGACCAACTTCTTTTCAGTAGAGTACAAGAACTTGTTACCTTCTGGACCACACAAAATAGCCATAGGTTCACCCAACAAAGAGGTTCTGAAAACCTTTGGGCTGTACTTTTCTTTACGGTACTTGACGAAATTTTCAGAAACACCGGACTTAACGCAGGACAAATAGTTCAAAGTTTCACCAATCAATGGCCAACCAGTTTGACCTGGTGGCAATGGAGCTTTAGAATCGTTCTCTTTTTGTGGGATTCTCTTGGTGTAAGAGAAGTAAGCAAACAACAGCAACAGTGGCAACAACAACAAAGACAGAGAGAAGAACAGGACCAT

> *AtCPR1*

TTACCAAACATCTCTCAAATATCTACCTTCAGTTTGCAACTTTTTAACAATAGCTTCAGCTTCACTAGAAGAAACACCTTCTTGTTCTTGGACAATAGTATGCAATGTTCTATGAACATCTCTAGCCATACCTTTAGCATCACCACAAACGTACAAATAACCTTCTTCCTTTATCAAATCCCAAACTTGTGCGGCCTTTTCCATCATTTTGTGTTGAACATATTCTTTTTGAGCACCTTCTCTGGAAAAAGCCATAATCAATTCACTAATAACACCTTGATCAACGAAGTTGTTCAATTCGTCTTCGTAAATAAAATCCATTTGTCTGTTTCTACAACCAAAAAACAACAAAGAAGAACCCAATTCTTCACCATCTTCCTTCAAAGCCATTCTTTCTTGCAAAAAACCTCTAAATGGAGCCAAACCTGTACCTGGACCAACCATAACAATTGGTGTAGATGGATTACTTGGCAACTTGAAATTAGAAGCTCTAATAAAAATTGGAGCACCTGAACATTCATGAGATTTTTCAGCTGGAACGGCGTTCTTCATCCATGTAGAACAAACACCTTTGTGTATTCTACCAGTAGGAGTAGGACCATAGACCAAAGCTGAGGTAACATGAACTCTAGATGGAGCCAATCTAGGAGAAGAGGAAATAGAATAATATCTTGGTTGCAATCTTGGAGCAATTGCAGCAAAGAAAACACCCAATGGTGGTTTAGCACTTGGAAAGGCAGCCATAACTTCCAACAAAGATCTTTGTGATGCAACTATCCATTGACTATATTCATCTTTACCATCTGGACTAGTCAAATGTTTCAACTTTTCAGCTTCGGATGGTTCTGTAGCATAAGCTGCCAAAGCAACCAAAGCAGATTTTCTTGGTGGATTCAACAAATCTGCATATCTAGCCAAACCAGTACCCAAAGTACATGGACCTGGAAATGGTGGTGGAACAGCACTTTCTAATGGACTACCATCTTCTTTGTCAGCATGTATAGAAAAAACCAAATCCAAAGAGTGACCCAACAACTTACCTGCTTCTTCGACAATTTCAACATGATTTTCAGCATAAACACCAACGTGATCACCGGTTTCATAAGTAATACCAGTTCTAGAAATGTCGAATTCCAAATGTATACAAGATCTATCACTTTCATGTGTGTGCAATTCCTTTTGAACAGCAACATCAACTCTACATGGGTGATGGATATCAATAGTAGTATTACCATTTGCAACGTTACTTTCCATAGATTTTTGAGTAGTGAATCTAGGATCATGAGTAACTACTCTATATTCTGGAATTACAGCAGTATAAGGTGTAGCAACACTCTTATCATCTTCATCTTTCAACAACTTATCCAATTCAGACCACAATGATTCTTTCCATGCGTTAAAGTCATCTTCTATTGATTGATCATCATCACCCAAACCAACTTCTATCAATCTTTTAGCACCCTTTTTACACAATTCTTCATCCAATACGATACCGATCTTGTTAAAATGTTCATATTGTCTATTACCCAAAGCAAAAACACCATATGCCAATTGTTGCAACTTAATATCTCTTTCGTTTTCTTCAGTAAACCACTTATAAAATCTAGCAGCATTATCAGTTGGTTCACCGTCACCATAGGTGGCAACACAAAAAAAAGCCAAGGTTTCCTTCTTCAATTTTTCTTCATATTGATCATCATCTGCTGCATAATCGTCCAAATCAATAACTTTAACAGCAGCCTTTTCATATCTAGCCTTAATTTCTTCAGACAATGCTTTAGCAAAACCTTCAGCAGTACCTGTTTGGGTACCGAAAAATATACTAACTCTAGTTTTACCAGAACCCAAATCCAAATCATCATCTTCGTCTTTAGCCATCAAAGATTTAGGAATCATCAAAGGCTTCAATTCACCACTTCTATCAGCAGTAGTTTTCTTCC

**Reference**

1. Dai ZB, Liu Y, Zhang XA, Shi MY, Wang BB, Wang D, Huang LQ, Zhang XL. Metabolic engineering of *Saccharomyces cerevisiae* for production of ginsenosides. Metab Eng. 2013; **20:**146-156.

2. Dai ZB, Wang BB, Liu Y, Shi MY, Wang D, Zhang XA, Liu T, Huang LQ, Zhang XL. Producing aglycons of ginsenosides in *bakers*' yeast. Sci Rep. 2014; **4:**3698.

3. Liang HC, Hu ZF, Zhang TT, Gong T, Chen JJ, Zhu P, Li Y, Yang JL. Production of a bioactive unnatural ginsenoside by metabolically engineered yeasts based on a new UDP-glycosyltransferase from *Bacillus subtilis*. Metab Eng. 2017; **44:**60-69.

4. Zhao FL, Bai P, Liu T, Li DS, Zhang XM, Lu WY, Yuan YJ. Optimization of a cytochrome P450 oxidation system for enhancing protopanaxadiol production in *Saccharomyces cerevisiae*. Biotechnol Bioeng. 2016; **113:**1787-1795.

5. Zhao FL, Du YH, Bai P, Liu JJ, Lu WY, Yuan YJ. Enhancing *Saccharomyces cerevisiae* reactive oxygen species and ethanol stress tolerance for high-level production of protopanoxadiol. Bioresour Technol. 2017; **227:**308-316.

6. Zhao FL, Bai P, Nan WH, Li DS, Zhang CB, Lu CZ, Qi HS, Lu WY. A modular engineering strategy for high-level production of protopanaxadiol from ethanol by *Saccharomyces cerevisiae*. AlChE J. 2019; **65:**866-874.

7. Kim JE, Jang IS, Sung BH, Kim SC, Lee JY. Rerouting of NADPH synthetic pathways for increased protopanaxadiol production in *Saccharomyces cerevisiae*. Sci Rep. 2018; **8:**15820.

8. Wang PP, Wei W, Ye W, Li XD, Zhao WF, Yang CS, Li CJ, Yan X, Zhou ZH. Synthesizing ginsenoside Rh2 in *Saccharomyces cerevisiae* cell factory at high-efficiency. Cell Discovery. 2019; **5:**5.

9. Kim JE, Jang IS, Son SH, Ko YJ, Cho BK, Kim SC, Lee JY. Tailoring the *Saccharomyces cerevisiae* endoplasmic reticulum for functional assembly of terpene synthesis pathway. Metab Eng. 2019; **56:**50-59.
